# Isolation and LC-QToF Characterization of Secondary Metabolites from an Endemic Plant *Artemisia heptapotamica* Poljak

**DOI:** 10.3390/molecules28072908

**Published:** 2023-03-23

**Authors:** Umit Mukatay, Mamdouh Nabil Samy, Bharathi Avula, Kumar Katragunta, Moldir Kemelbek, Azhar Zhubanova, Ikhlas A. Khan, Samir Anis Ross

**Affiliations:** 1 Department of Biology and Biotechnology, Al-Farabi Kazakh National University, Almaty 050040, Kazakhstan; umit.muhatai@gmail.com (U.M.); a.zhubanova@kaznu.kz (A.Z.); 2 National Center for Natural Products Research, School of Pharmacy, University of Mississippi, Oxford, MS 38677, USA; mamdouh.eskandr@mu.edu.eg (M.N.S.); bavula@olemiss.edu (B.A.); kkatragu@olemiss.edu (K.K.); moldir.kemelbekk@gmail.com (M.K.); khan@olemiss.edu (I.A.K.); 3 Department of Pharmacognosy, Faculty of Pharmacy, Minia University, Minia 61519, Egypt; 4 Department of Chemistry and Chemical Technology, Al-Farabi Kazakh National University, Almaty 050040, Kazakhstan; 5 Department of BioMolecular Science, Division of Phamacognosy, School of Pharmacy, University of Mississippi, Oxford, MS 38677, USA

**Keywords:** Asteraceae, *Artemisia heptapotamica*, LC-QTpoF, sesquiterpene lactones, flavonoids, coumarate derivatives, aliphatic compounds

## Abstract

Phytochemical investigation of the aerial parts of *Artemisia heptapotamica* Poljak led to the isolation of ten known compounds, including four alkyl *p*-coumarates: octadecyl *trans*-*p*-coumarate (**1**), icosy *trans*-*p*-coumarate (**2**), docosyl *trans*-*p*-coumarate (**3**), and tetracosyl *trans*-*p*-coumarate (**4**), one sesquiterpene lactone: santonin (**5**), four flavonoids; axillarin (**6**), quercetin 3-*O*-methyl ether (**7**), luteolin (**8**), and quercetin (**9**), and one phenolic acid derivative: *p*-coumaric acid (**10**). The structures of the isolated compounds were identified by various spectroscopic analyses. Additionally, the antimicrobial activity of the total extract and different fractions was screened, and they exhibited no inhibition of the growth of *Candida albicans*, *C. neoformans*, *Aspergillus fumigatus*, methicillin-resistant *Staphylococcus aureus* (MRS), *E. coli*, *Pseudomonas aeruginosa*, *Klebsiella pneumonia*, and Vancomycin-resistant *Enterococci* (VRE) at the tested concentrations ranging from 8 to 200 μg/mL. The identification and tentative characterization of the secondary metabolites were conducted using LC-QToF analysis. This method helps in the putative characterization of sesquiterpene lactones, flavonoids, coumarate derivatives, and aliphatic compounds. The developed method identified 43 compounds, of which the majority were sesquiterpene lactones, such as eudesmanolides, germacranolides, and guaianolide derivatives, followed by flavonoids. The proposed LC-QToF method helps develop dereplication strategies and understand the major class of chemicals before proceeding with the isolation of compounds.

## 1. Introduction

*Artemisia* L. is the largest genus belonging to the family Asteraceae [1]. This genus is known for its essential oils with aromatic and medicinal properties, which are used in traditional medicine as well as in modern scientific medicinal practices [2,3]. Wormwood is widespread and widely found across geographical areas: in the temperate zone of Eurasia, North and South Africa, Europe, the Middle East, Afghanistan, Pakistan, China, Korea, Japan, and India (Himalayas). Plenty of species are found in Russia (174 species), mainly in Yakutia (22), Siberia (70), and Buryatia (46), and also in China (200) [4]. Approximately 500 species of wormwood are known worldwide, and 81 species grow in Kazakhstan. However, only 30 of Kazakhstan’s wormwood species have been studied from various biological, ecological, and chemical perspectives. [5]. Extracts of *Artemisia* species improve digestion, stimulate appetite, and are used to treat dyspepsia, acid gastritis, gastrointestinal tract diseases, liver diseases, gall bladder problems, insomnia, malaria, influenza, and upper respiratory tract ailments. They have also been used to treat bronchial asthma, rheumatism, eczema, dysentery, anemia, jaundice, obesity, meteorism, migraine, hypertension, and tuberculosis. *Artemisia* species have been found to have various pharmacological activities, such as anthelmintic, antimicrobial, anti-inflammatory, antitumor, antioxidant, cytostatic, antifungal, antimalarial, antileishmaniasis, antinociceptive, immunomodulatory, and antipyretic activity, as well as potent inhibitory activity against FPTase [6]. One of the endemic *Artemisia* plants is *Artemisia heptapotamica* Poljak. There are few chemical studies on this plant, and a recent study showed the presence of methyl ether of quercetin [7], in addition to monomeric and dimeric sesquiterpene lactones from this plant. Most isolated monomeric sesquiterpenes showed strong inhibition of the lipopolysaccharide (LPS)-induced NF-κB activation in a THP1-Dual cell model [8]. This study aimed to isolate the compounds from the whole plant and characterize them using NMR and LC-QToF analysis, in addition to evaluation of the antimicrobial activity of the total extract and different fractions.

## 2. Results and Discussion

### 2.1. Identification of the Isolated Compounds

**Figure 1:**
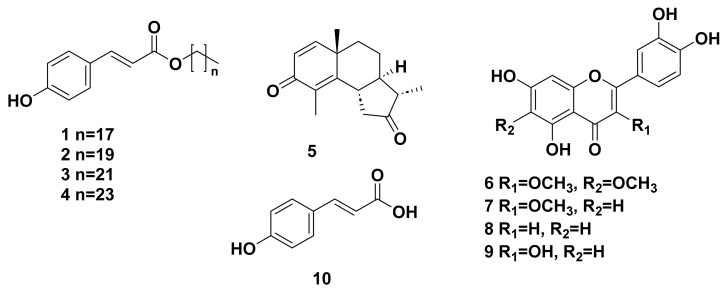
Structures of the isolated compounds from *A. heptapotamica*.

The ethyl acetate fraction of methanol of *A. heptapotamica* was fractionated and purified using silica gel column chromatography, producing ten known compounds (Figure 1), including four alkyl *p*-coumarates: octadecyl *trans*-*p*-coumarate (**1**), icosy *trans*-*p*-coumarate (**2**), docosyl *trans*-*p*-coumarate (**3**), and tetracosyl *trans*-*p*-coumarate (**4**) [9]; one sequiterpene: santonin (**5**) [10,11]; four flavonoids: axillarin (**6**) [12], quercetin 3-*O*-methyl ether (**7**) [13], luteolin (**8**) [14], and quercetin (**9**) [15]; and one phenolic acid derivatives: *p*-coumaric acid (**10**) [16]. The structures of the isolated compounds were elucidated using different spectroscopic analyses such as 1D NMR experiments (^1^H, ^13^C, DEPTQ, and DEPT), HR-ESI-MS analysis (Supplementary Materials: Figures S1–S27), and comparison with the published data. Compounds **1**–**10** were isolated from *A. heptapotamica* for the first time.

It was clear that the fraction of alkyl *p*-coumarate did not correspond to any single compound. Thus, it was subjected to a detailed study using ^1^H NMR, DEPTQ, and HR-ESI-MS to identify the structures of these compounds in a mixture.

### 2.2. Antimicrobial Activity

The antibacterial and antifungal activities of the total extract, as well as *n*-hexane and EtOAc fractions of the aerial parts of *A. heptapotamica*, were studied. They exhibited no inhibition of the growth of *Candida albicans*, *C. neoformans*, *Aspergillus fumigatus*, methicillin-resistant *Staphylococcus aureus* (MRS), *E. coli*, *Pseudomonas aeruginosa, Klebsiella pneumonia*, and Vancomycin-resistant *Enterococci* (VRE) at the tested concentration ranging from 8 to 200 μg/mL. Although different studies have shown that quercetin, its derivatives, and luteolin had broad-spectrum antibacterial and antifungal properties [17,18], the methanolic extract and different fractions displayed no activity on the tested microorganisms due to the small concentration used in the assay.

### 2.3. Identification and Tentative Characterization of Secondary Metabolites Using LC-QToF

**Figure 2:**
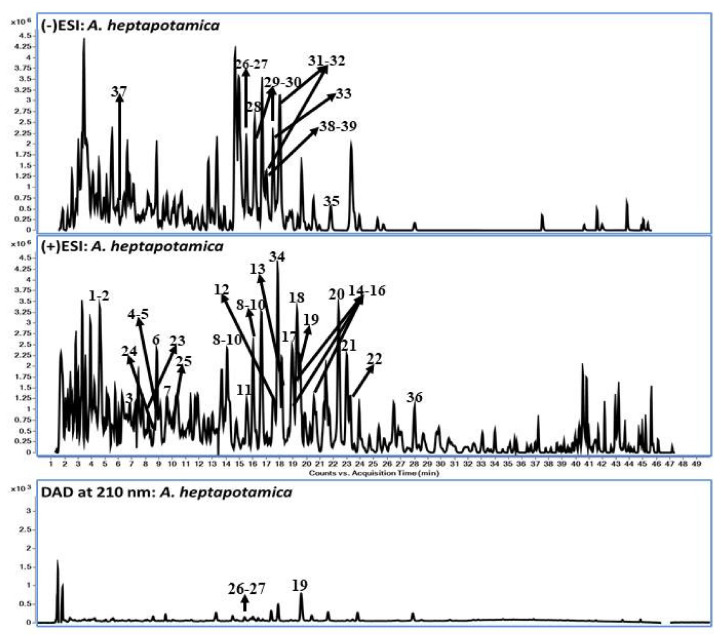
LC-DAD-QToF chromatograms of *Artemisia heptapotamica* aerial part: QToF-MS Base Peak chromatograms in negative and positive modes and DAD chromatogram at 210 nm.

The secondary metabolites from aerial parts of *A. heptapotamica* (methanolic extract) were separated using liquid chromatography, followed by their characterization using time-of-flight mass spectrometry. The mass accuracy for the putatively identified compounds was less than 4 ppm error. The identified compounds presented in Table 1 consisted of sesquiterpene lactones and flavonoids in the majority. In addition, coumarate derivatives and *p*-coumaric acid were also identified. Sesquiterpene lactones were detected in an ESI-positive ionization mode with [M + NH_4_]^+^ and [M + Na]^+^ adduct precursor ions. The tentative characterization of compounds was processed based on the molecular features, such as accurate mass, fragment ions (neutral ions), and precursor ion molecular formula. The representative base peak chromatograms (BPC) in negative and positive modes, along with LC-DAD profiles at 210 nm, are shown in Figure 2.

#### 2.3.1. Sesquiterpene Lactones (**1**–**22**)

Sesquiterpene lactones are the major class of compounds in the *Artemisia* species. To perform characterization using LC-QToF, a few sesquiterpene lactones, e.g., santonin (compound **19**), were isolated from aerial parts of *A. heptapotamica* in this study. Santonin mass fragmentation initiated with loss of water molecules, resulting in *m/z* 228.1219 [M + H-H_2_O]^+^. The following loss of -CO and sequential loss of -C_2_H_4_ and -CH_4_ resulted in *m/z* 201.1271, 173.0954, and 157.0648, respectively. Further loss of -CO ion resulted in the opening of the lactone moiety. Further loss of ketene moiety (-C_2_H_2_O) from the opened six-membered ring resulted in the formation of *m/z* 115.0542. Fragments with the least molecular weight (*m/z* 105.0698 and 91.0544) were useful in determining the backbone skeleton of the molecule. These characteristic fragments matched with the reported santonin fragmentation pathway [19]. Furthermore, based on the high-resolution mass spectrometric data, corresponding fragment ions of different types of sesquiterpene backbone skeletons were observed, i.e., eudesmanolides, germacranolides, and guianolide derivatives. The molecular features depicted in Table 1 provided further confirmation of the various sesquiterpene moieties. A total of twenty-two compounds of sesquiterpene lactones were tentatively characterized. The identified compounds were reported in various *Artemisia* species. The *m/z* values of the tentatively characterized sesquiterpenes are shown in Table 1 under the guidance of reported mass fragments and isolation reports (from the dictionary of natural products) [20,21,22,23,24]. Based on the fragmentation pattern of santonin, remaining sesquiterpene lactones were identified and tentatively characterized.

#### 2.3.2. Flavonoids (**23**–**36**)

Using the isolated reference compounds and exact mass measurements, fourteen flavonoid compounds were identified. Most of the compounds are flavone derivatives. There are two quercetin derivatives, along with quercetin and luteolin. Flavonoids have established mass fragmentation patterns based on the literature previously reported [20,22,23,24,25]. The flavone derivatives showed that the fragmentation starts with the loss of functional groups, such as water, methoxy group, and -glc. The aglycone molecular weight helps in understanding the backbone skeleton. The corresponding flavonoids’ exact mass [M + H]^+^/[M-H]^−^ and fragment ions are shown in Table 1. Compound **25** showed *m/z* 509.1284 [M + H]^+^ with a C_23_H_24_O_13_ molecular formula. The corresponding fragment ions resulted in *m/z* 347.0762 [M + H-Glc]^+^, 331.0434, and 289.0329, respectively. The compound was characterized as 3,4,5,7-tetrahydroxy-3-methoxyflavone 7-O-β-D-glucopyranoside. Similarly, the flavone derivatives were characterized based on their fragmentation pathways. Along with hydroxy/methoxy flavone derivatives, simple aglycone flavonoids such as quercetin (compound **27**) and luteolin (compound **26**) were identified. Quercetin showed *m/z* 303.0491 [M + H]^+^ and *m/z* 301.0354[M-H]^−^ with fragment ions at *m/z* 153 and 151, respectively. Furthermore, luteolin showed *m/z* 287.0548 [M + H]^+^ and 285.0406 [M-H]^−^ in both positive and negative ionization modes. In addition, one quercetin derivative (compound **33**) was identified with *m/z* 317.0658 [M + H]^+^ with fragment ions at *m/z* 301.0343 (such as quercetin aglycone), 274.0465, and 137.0233. The compound was characterized as quercetin 3-*O*-methyl ether.

#### 2.3.3. Others (**37**–**43**)

Seven compounds were identified apart from sesquiterpenes and the flavonoid class of compounds. At 5.8 min, the chromatographic peak showed *m/z* 165.0546 [M + H]^+^ and 163.0398 [M-H]^−^ with fragment ions at *m/z* 119.0502 [M-H-CO_2_]^−^ in the negative mode of ionization. The compound was characterized as *p*-coumaric acid (compound **37**). In addition, an aliphatic compound was identified with chemical formula C_10_H_18_O_3_. Compound **38** was tentatively characterized based on the exact mass at m/z 185.1186 [M-H]^−^ and corresponding fragment ion at *m/z* 167.1077 [M-H-H_2_O]^−^. Compound **39** was identified as 3,4,5-tri-caffeoylquinic acid with a precursor ion at *m/z* 677.1524 [M-H]^−^. The corresponding fragment ions were at *m/z* 515.1198 [M-H-Glc]^−^, 353.0822 [M-H-Glc-Glc]^−^, and 191.0572 [quinic acid] [26], respectively. In addition, non-polar compounds (compounds **40**–**43**) were matched with isolated standards. The corresponding adduct ions in the negative mode, along with their retention times, are listed in Table 1.

The isolated compounds, as well as chromatographic peaks with distinctive fragment ions, were confirmed based on the reported literature studies whose corresponding references are listed in Table 1. Furthermore, compounds whose fragment ions were not observed in this study were tentatively identified based on their molecular features and database searches such as from the dictionary of natural products and other literature of isolated compounds from *Artemisia* species.

## 3. Materials and Methods

### 3.1. General Experimental Procedures

^1^H and ^13^C NMR spectra were recorded on a Bruker Avance 400 MHz instrument. HR-ESI-MS was taken on BrukerBioApex-FTMS with electron spray ionization. Solvents used in this work, e.g., *n*-hexane, dichloromethane (DCM), ethyl acetate (EtOAc), methanol (MeOH), and ethanol (EtOH), were purchased from Fisher Scientific, USA. Deuterated solvents purchased from Cambridge Isotope Laboratories, Inc., Tewksbury, MA, USA, including methanol-d_4_ (CD_3_OD), chloroform-d_3_ (CDCl_3_), and pyridine-d_5_ (C_5_H_5_N-d_5_), were used for nuclear magnetic resonance (NMR) spectroscopic analyses. Acetonitrile, methanol, and formic acid of HPLC-certified grade were used for LC-MS analysis, and water was purified using a Milli-Q system (Millipore, Bedford, MA, USA). Column chromatography (CC) was performed using silica gel 60 (Merck, Darmstadt, Germany; 70–230 mesh). Thin-layer chromatography (TLC) analyses were carried out using pre-coated silica G plates w/UV254 (Sorbent Technologies, USA; 20 × 20 cm, 200 µm in thickness). An ultraviolet lamp (Spectroline ENF-240C, Spectronics Corporation, New York, NY, USA) was used for visualization of spots on thin-layer chromatograms at 254 and/or 365 nm. Spots were visualized by spraying with 2% vanillin (Tokyo Chemical Industry Co. Ltd., Tokyo, Japan) in sulfuric acid–ethanol followed by heating at 110 °C.

### 3.2. Plant Material

The whole plant of *A. heptapotamica* was collected in October 2021 from Kokpek village, Almaty, Kazakhstan. Identification and authentication were performed by Dr. Danilov Mikhail Petrovich. The sample was stored with the voucher number 0000723 in the Main Botanical Garden of the Institute of Botany and Phyto-introduction, Almaty, Kazakhstan. The same sample was assigned with the NCNPR number 25173 and stored in the National Center for Natural Product Research Botanical Repository, University of Mississippi, USA.

### 3.3. Extraction and Isolation

The collected aerial parts of *A. heptapotamica* were first dried in the shade and then crushed into small pieces. The dried plant material (1.65 kg) was extracted by maceration with 95% methanol three times at room temperature and was concentrated under reduced pressure to yield 253.35 g of the total extract. The total extract was mixed with a small amount of distilled water and successively fractionated with *n*-hexane and ethyl acetate. The fractions were concentrated under reduced pressure to produce *n*-hexane (29.1 g) and ethyl acetate fractions (58.74 g).

The EtOAc (58.74 g) fraction was subjected to fractionation using VLC silica gel CC using *n*-hexane and EtOAc (10:0, 75:25, 50:50, 25:75, and 0:10) and finally washed with MeOH, affording 6 fractions. Fraction F-4 (395.9 mg) was subjected to silica gel CC (40 × 2 cm, 30 g), using DCM–MeOH gradient mixtures to increase the polarity gradually in 2% MeOH till 80 % MeOH and finally 100% MeOH were produced, yielding 37 subfractions. The seventeenth subfraction (21.1 mg) afforded a mixture of compounds **3** and **4**. The eighteenth subfraction (12.9 mg) produced a mixture of compounds **2**–**4**. The nineteenth subfraction (16.5 mg) produced a mixture of compounds **2**–**4**. The twentieth subfraction (6.2 mg) furnished a mixture of compounds **1**–**4**.

Fraction F-5 (14.3 g) was subjected to silica gel CC (75 × 4 cm, 375 g), using DCM–MeOH gradient mixtures to gradually increase the polarity in 2% MeOH till 80% and finally 100% MeOH were produced, affording 29 subfractions. The eleventh subfraction was crystalized to produce compound **5** (45 mg). The eighteenth subfraction was precipitated to give compound **6** (27 mg). Subfraction F-5-21 (1.26 g) was purified on silica gel CC using DCM–MeOH gradient mixtures to gradually increase the polarity in 2% MeOH till 80% MeOH was produced, affording compound **7** (8.4 mg). Subfraction F-5-24 (342.8 mg) was rechromatographed over silica gel CC using DCM–MeOH gradient mixtures to gradually increase the polarity in 2% MeOH till 80% MeOH was reached, producing a mixture of compounds **7** and **8** (14.5 mg) and **9** (24.4 mg). Subfraction F-5-26 (450.1) was purified on silica gel CC using DCM–MeOH gradient mixtures to gradually increase the polarity in 2% MeOH till 80% MeOH was reached, producing compound **10** (8.2 mg).

### 3.4. Evaluation of Antimicrobial Activity

The antimicrobial activity of the total extract and different fractions was evaluated using the method reported by Samy et al. [32].

### 3.5. Liquid Chromatography-Diode Array Detector-Quadrupole Time-of-Flight Mass Spectrometry (LC-DAD-QToF)

About 25 mg of extract was sonicated in 1.0 mL of methanol for 5 min, followed by centrifugation for 15 min at 7000 rpm. The clear filtered supernatant solution was used for analysis.

The liquid chromatographic system was an Agilent Series 1290, and separation was achieved on an Acquity UPLC^TM^ HSS C18 column (100 mm × 2.1 mm I.D., 1.8 µm). The mobile phase consisted of water with 0.1% formic acid (A) and acetonitrile with 0.1% formic acid (B) at a flow rate of 0.23 mL/min. Analysis was performed using the following gradient elution: 15% B to 40% B in 30 min, then to 100% B in the next 15 min. Each run was followed by a 5 min wash with 100% B and an equilibration period of 15 min with 85% A/15% B. Two microliters of the sample were injected. The column temperature was 40 °C.

The mass spectrometric analysis was performed with a QToF-MS-MS (Model #G6545B, Agilent Technologies, Santa Clara, CA, USA) equipped with an ESI source with Jet Stream technology, using the following parameters: drying gas (N_2_) flow rate, 13 L/min; drying gas temperature, 325 °C; nebulizer pressure, 30 psi; sheath gas temperature, 300 °C; sheath gas flow, 11 L/min; capillary voltage, 3500 V; nozzle voltage, 0 V; skimmer, 65 V; Oct RF V, 750 V; and fragmentor voltage, 125 V. All the operations, acquisition of data, and analysis of data were controlled using Agilent MassHunter Acquisition Software ver. A.10.1 and processed with MassHunter Qualitative Analysis software ver. B.07.00. Each sample was analyzed in positive and negative modes over the range of *m/z* 50–1700 and an extended dynamic range. Accurate mass measurements were obtained by employing ion correction techniques using reference masses at *m/z* 121.0509 (protonated purine) and 922.0098 (protonated hexakis [1H, 1H, 3H-tetrafluoropropoxy] phosphazine or HP-921) in positive ion mode, while *m/z* 112.9856 (deprotonated trifluoroacetic acid-TFA) and 1033.9881 (TFA adducted HP-921) were used in negative ion mode. Samples were analyzed in all-ion MS–MS mode, where experiment 1 was carried out with a collision energy of zero and experiment 2 with a fixed collision energy of 45 eV.

## 4. Conclusions

Ten compounds were isolated from the ethyl acetate fraction of *A. heptapotamica*, including four flavonoids, four alkyl coumarate, one sesquiterpene lactone, and one phenolic acid. Additionally, chemical characterization of the methanolic extract of *A. heptapotamica* using LC-QToF analysis led to the identification of 43 compounds, of which sesquiterpene lactones were the major secondary metabolites, followed by flavonoids. The chemical characterization of the methanolic extract of *A. heptapotamica* also showed the presence of the isolated compounds (**1**–**10**), which matched with the authentic samples of *A. heptapotamica*.

## Figures and Tables

**Table 1:** Tentative identification and characterization of phytochemical compounds in *Artemisia heptapotamica* whole plant extract using LC-QToF in positive and negative ionization modes.

#	RT (min)	Compound Name	Molecular Formula	Mass	Adduct (+ve Mode)	Fragment Ions (Positive Ion Mode)	Adduct (-ve Mode)	Fragment Ions (Negative Ion Mode)	Ref.
Sesquiterpene lactones									
**1**	4.6	Artemisinin/Artemisinin G	C_15_H_22_O_5_	282.1467	300.1809 (300.1813) * [M + NH_4_]^+^	181.0845, 105.0685, 91.0534	-	-	[22]
**2**	4.7						-	-	
**3**	6.8	Artelavanolide A/ Austroyunnane B/C/E/ Artemdubolide I	C_15_H_20_O_6_	296.1260	314.1584 (314.1598) [M + NH_4_]^+^	183.1003, 153.0895, 107.0845	-	-	[20]
**4**	8.7	Guaianolide derivative	C_15_H_18_O_4_	262.1205	263.1271 (263.1278) [M + H]^+^ 285.1095 (285.1097) [M + Na]^+^	245.1165, 233.1162, 91.0433, 772.0286	-	-	[20,27]
**5**	8.8	Rupicoline B/ Hydroxyachillin	C_15_H_20_O_4_	264.1362	265.1423 (265.1434) [M + H]^+^ 287.1242 (287.1254) [M + Na]^+^	-	263.1294 (263.1289) [M-H]^−^	-	[20]
**6**	8.9	Artemdubolide I	C_16_H_21_O_8_	296.1260	-	-	295.1177 (295.1187) [M-H]^−^ 341.1231 (341.1242) [M + COOH]^−^	-	[20]
**7**	9.3	Millifolide A	C_30_H_34_O_9_	538.2203	-	-	537.2119 (537.2130) [M-H]^−^ 583.2145 (583.2185) [M + COOH]^−^	-	[20]
**8**	14.1	Dihydroxy-eudesmen-olide/ Dihydroxy-germacradien-olide	C_15_H_22_O_4_	266.1518	267.1586 (267.1591) [M + H]^+^ 284.1845 (284.1856) [M + NH_4_]^+^	249.1470	-	-	[20,21]
**9**	16.1						-	-	
**10**	16.6						-	-	
**11**	15.5	Valerianin C	C_17_H_24_O_7_	340.1522	363.1403 (363.1414) [M + Na]^+^	323.1477, 305.1385, 281.1376, 169.1210, 151.1107, 109.1003	-	-	[20]
**12**	17.4	Ezoartemin/Yamayomoginin (Guaianolide derivative)	C_17_H_22_O_7_	338.1366	356.1695 (356.1704) [M + NH_4_]^+^	279.1582, 261.1459, 247.1307. 205.1198, 173.0940, 153.0892	-	-	[20,27]
**13**	18.1	9-Acetoxy-5-hydroperoxy-4(15),11(13)- eudesmadien-12-oic acid	C_17_H_24_O_6_	324.1573	342.1898 (342.1911) [M + NH_4_]^+^	281.1726, 265.1430	-	-	[20,21]
**14**	18.8	Trihydroxy-guaiadien-olide/ Eudesmanolide derivatives	C_15_H_20_O_5_	280.1311	298.1636 (298.1649) [M + NH_4_]^+^ 303.1199 (303.1203) [M + Na]^+^	263.1262, 155.1057, 109.1005	-	-	[20,21,27]
**15**	19.2						-	-	
**16**	20.4						-	-	
**17**	19.2	Ajaniaolide B	C_14_H_18_O_3_	234.1256	235.1318 (235.1329) [M + H]^+^		-	-	[20]
**18**	19.5	3β-Acetoxy-1β-hydroxyarbusculin	C_17_H_24_O_6_	324.1573	325.1640 (325.1646) [M + H]^+^ 347.1458 (347.1465) [M + Na]^+^	247.1314 [C_15_H_18_O_3_ + H]^+^ (arbusculin skeleton), 173.0947, 135.0794, 115.0538	-	-	[28]
**19**	19.7	Santonin	C_15_H_18_O_3_	246.1256	247.1329 (247.1329) [M + H]^+^	229.1219 [M + H-H_2_O]^+^, 201.1271 [M + H-H_2_O-CO]^+^, 173.0954 [M + H-H_2_O-CO-C_2_H_4_]^+^, 157.0648 [M + H-H_2_O-CO-C_2_H_4_-CH_4_]^+^, 129.0700 [M + H-H_2_O-CO-C_2_H_4_-CH_4_-CO]^+^, 115.0542 [M + H-H_2_O-CO-C_2_H_4_-CH_4_-C_2_H_2_O]^+^, 105,0698 [C_8_H_8_ + H]^+^, 91.0544 [C_7_H_6_]^+^	-	-	[19]
**20**	22.4	Arbusculin C/Taurin/Finitin	C_15_H_20_O_3_	248.1412	271.1305 (271.1295) [M + Na]^+^	231.1363, 141.0685, 128.0606, 115.0530	-	-	[20,28]
**21**	23.0	3-Acetyldihydroridentin/Nitrosin /Torrentin/Epitorrentin/Herbolide B/C/D/	C_17_H_24_O_5_	308.1624	331.1505 (331.1516) [M + Na]^+^	291.1577, 249.1484, 231.1363, 105.0695, 91.0537	-	-	[20]
**22**	23.3	Dihydroeudesmanomolide	C_19_H_26_O_7_	366.1679	389.1577 (389.1571) [M + Na]^+^	229.1203, 135.1152	-	-	[20,21]
**Flavonoids**									
**23**	8.1	3,3′,4′,5,7-pentahydroxy-6-methoxyflavone; 3-*O*-[α-L-rhamnopyranosyl-(1 → 6)-β-D-glucopyranoside]	C_28_H_32_O_17_	640.1639	641.1703 (641.1712) [M + H]^+^	347.0745	-	-	[20]
**24**	8.5	3′,4′,5,7-tetrahydroxy-3-methoxyflavone; 7-*O*-β-*D*-glucopyranoside	C_22_H_22_O_12_	478.1111	479.1176 (479.1184) [M + H]^+^	302.0405	-	-	[20]
**25**	9.6	3′,4′,5,7-tetrahydroxy-3,6-dimethoxyflavone; 7-*O*-β-*D*-glucopyranoside	C_23_H_24_O_13_	508.1217	509.1284 (509.1290) [M + H]^+^	347.0762, 331.0434, 314.0487, 289.0329, 105.0687, 91.0534	507.1149 (507.1144) [M-H]^−^	492.0915, 345.0626, 329.0311, 314.0069	[20]
**26**	15.3	Luteolin	C_15_H_10_O_6_	286.0477	287.0548 (287.0550) [M + H]^+^	-	285.0406 (285.0405) [M-H]^-^	-	[29]
**27**	15.4	Quercetin	C_15_H_10_O_7_	302.0427	303.0491 (303.0499) [M + H]^+^	153.0145	301.0354 (301.0356) [M-H]^−^	151.0033	[21]
**28**	15.9	Tetrahydroxy-methoxyflavone	C_16_H_12_O_7_	316.0583	317.0652 (317.0656) [M + H]^+^	274.0463, 168.0054, 140.0100	315.0516 (315.0510) [M-H]^−^	-	[29]
**29**	16.1	3′,4′,5,5′,7-pentahydroxyflavone; 3′-Me ether	C_16_H_12_O_7_	316.0583	-	-	315.0511 (315.0510) [M-H]^−^	300.0279, 271.0250, 243.0304, 227.0349	[25,29]
**30**	17.4								
**31**	16.5	3′,4′,5,6-tetrahydroxy-3,7-dimethoxyflavone	C_17_H_14_O_8_	346.0689	-	-	345.0619 (345.0616) [M-H]^−^	287.0195, 149.0246	[25,29]
**32**	18.0								
**33**	17.3	Quercetin 3-*O*-methyl ether	C_16_H_12_O_7_	316.0583	317.0658 (317.0656) [M + H]^+^	301.0343, 274.0465, 137.0233	315.0512 (315.0510) [M-H]^−^	300.0277, 271.0250, 243.0307, 227.0348, 199.0398	[21]
**34**	17.8	Axillarin	C_17_H_14_O_8_	346.0689	347.0762 (347.0761) [M + H]^+^	289.0336, 269.0439, 203.0335, 137.0232	-	-	[29]
**35**	21.7	3′,4′,5′,6,7-pentahydroxyflavone; 3′,5′-dimethyl ether	C_17_H_14_O_7_	330.0740	-	-	329.0670 (329.0667) [M-H]^−^	271.0252	[25,29]
**36**	28.0	Dihydroxy-trimethoxyflavone	C_18_H_16_O_7_	344.0896	345.0961 (345.0969) [M + H]^+^	329.0652, 287.0536, 269.0436, 169.0129	-	-	[25,29]
**Others**									
**37**	5.8	*p*-Coumaric acid	C_9_H_8_O_3_	164.0473	(165.0546) 165.0546 [M + H]^+^	-	163.0398 (163.0401) [M-H]^−^	119.0502	[21]
**38**	16.7	6-Methyl-2-methylene-6-octene-triol	C_10_H_18_O_3_	186.1256	-	-	185.1186 (185.1183) [M-H]^−^	167.1077	[20]
**39**	16.9	3,4,5-tri-*O*-caffeoylquinic acid	C_34_H_30_O_15_	678.1585	-	-	677.1524 (677.1512) [M-H]^−^	515.1198, 409.0245, 353.0822, 329.0673, 285.0407, 271.0257, 243.0302, 191.0571, 179.0351, 173.0458, 161.0243	[22]
**40**	46.2	Docosyl *p*-coumarate	C_31_H_52_O_3_	472.3916	-	-	471.3848 (471.3844) [M-H]^−^	-	[30,31]
**41**	49.1	Icosy *p*-coumarate	C_29_H_48_O_3_	444.3603	-	-	443.3536 (443.3531) [M-H]^−^	-	[30,31]
**42**	52.2	Octadecyl *p*-coumarate	C_27_H_44_O_3_	416.3290	-	-	415.3226 (415.3218) [M-H]^−^	-	[30,31]
**43**	56.5	Tetracosyl *p*-coumarate	C_33_H_56_O_3_	500.4229	-	-	499.4159 (499.4157) [M-H]^−^	-	[30,31]

* Theoretical mass.

## Data Availability

Not applicable.

## Supplementary Materials

The following supporting information can be downloaded at: https://www.mdpi.com/article/10.3390/molecules28072908/s1, Scheme S1: Structures of the isolated compounds from *A.* heptapotamica; Figures S1–S27: 1H, 13C, and DEPT NMR and HR-ESI-MS spectra of compounds **1**–**10**.

## Sample Availability

No samples of the compounds are available from the authors.
