# Supplementary material for: Isolation and LC-QToF Characterization of Secondary Metabolites from an Endemic Plant Artemisia heptapotamica Poljak
Source: Molecules. 2023 Mar 23;28(7):2908. doi: 10.3390/molecules28072908 (PMC10096343; doi:10.3390/molecules28072908)
Supplement: Supplementary file 1 [file molecules-28-02908-s001.zip › molecules-2283955-supplementary.pdf]

Supplementary Material

Isolation and LC-QToF Characterization of Secondary Metabolites from an Endemic Plant *Artemisia heptapotamica* Poljak

Umit Mukatay [1,2], Mamdouh Nabil Samy [2,3], Bharathi Avula [2], Kumar Katragunta [2], Moldir Kemelbek [2,4], Azhar Zhubanova [1], Ikhlas A. Khan [2] and Samir Anis Ross [2,4,5,*]

[1] Department of Biology and Biotechnology, Al-Farabi Kazakh National University, Almaty 050040, Kazakhstan; umit.muhatai@gmail.com (U.M.); a.zhubanova@kaznu.kz (A.Z.)

[2] National Center for Natural Products Research, School of Pharmacy, University of Mississippi, Oxford, MS 38677, USA; mamdouh.eskandr@mu.edu.eg (M.N.S.); bavula@olemiss.edu (B.A.); kkatragu@olemiss.edu (K.K.); moldir.kemelbekk@gmail.com (M.K.); khan@olemiss.edu (I.A.K.)

[3] Department of Pharmacognosy, Faculty of Pharmacy, Minia University, Minia 61519, Egypt

[4] Department of Chemistry and Chemical Technology, Al-Farabi Kazakh National University, Almaty 050040, Kazakhstan

[5] Department of BioMolecular Science, Division of Phamacognosy, School of Pharmacy, University of Mississippi, University, MS 38677, USA

* Correspondence: sross@olemiss.edu

Abstract: Phytochemical investigation of the aerial parts of Artemisia heptapotamica Poljak. led to isolation of ten known compounds, including four alkyl *p*-coumarates: octadecyl *trans-p*-coumarate (**1**), icosy *trans-p*-coumarate (**2**), docosyl *trans-p*-coumarate (**3**), and tetracosyl *trans-p*-coumarate (**4**), one sequiterpene lactone: santonin (**5**), four flavonoids; axillarin (**6**), quercetin 3-*O*-methyl ether (**7**), luteolin (**8**), and quercetin (**9**), and one phenolic acid derivative: *p*-coumaric acid (**10**). The structures of the isolated compounds were identified by various spectroscopic analyses. Additionally, the antimicrobial activity of the total extract and different fractions was screened, and they exhibited no inhibition of the growth of *Candida albicans*, *C. neoformans*, *Aspergillus fumigatus*, methicillin-resistant *Staphylococcus aureus* (MRS), *E. coli*, *Pseudomonas aeruginosa*, *Klebsiella pneumonia*, and Vancomycin-resistant *Enterococci* (VRE) at the tested concentration ranging from 8 to 200 μg/mL. The identification and tentative characterization of the secondary metabolites were investigated using LC-QToF analysis. This method helps in putative characterization of sesquiterpene lactones, flavonoids, coumarate derivatives and aliphatic compounds. The developed method was able to identify 43 compounds of which majority were sesquiterpene lactones such as eudesmanolides, germacranolides and guaianolide derivatives, followed by flavonoids. The proposed LC-QToF method helps in dereplication strategies and understand the major class of chemistry before proceeding for isolation of compounds.

Keywords: Asteraceae; *Artemisia heptapotamica*; LC-QToF; sesquiterpene lactones; flavonoids; coumarate derivatives; aliphatic compounds

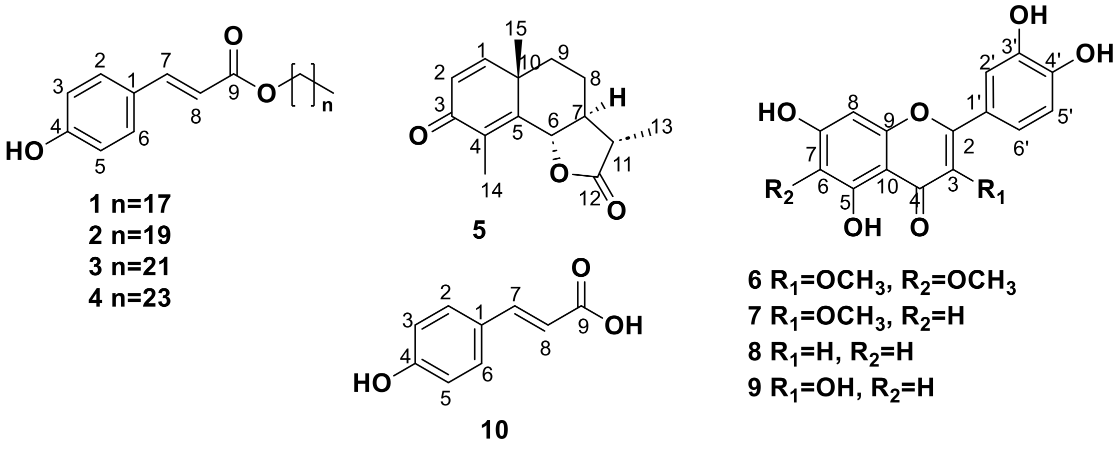

Scheme S1. Structures of the isolated compounds from *A. heptapotamica*.

Octadecyl *trans-p*-coumarate (1): ^{1}H-NMR (pyridine-d_5, 400 MHz) δ_H : 0.87 (3H, t, J=6.9 Hz, Me-18′), 1.27— 1.41 (30H, m, H_2-3′— H_2-17′), 1.73 (2H, m, H_2-2′), 4.30 (2H, t, J=6.9 Hz, H_2-1′), 6.62 (1H, d, J=15.9 Hz, H-8), 7.15 (2H, d, J=8.4 Hz, H-3,5), 7.57 (2H, d, J= 8.4 Hz, H-2,6), 7.96 (1H, d, J=15.9 Hz, H-7). ^{13}C-NMR (pyridine-d_5, 100 MHz) δ_C : 14.1 (C-18′), 22.7 (C-17′), 26.1 (C-16′), 29.0, 29.4 — 29.8 (C-3′— C-15′), 31.9 (C-2′), 64.3 (C-1′), 115.0 (C-8), 116.6 (C-3,6), 125.9 (C-1), 130.5 (C-2,6), 144.9 (C-7), 161.3 (C-4), 167.2 (C-9). HR-ESI-MS (negative-ion mode) m/z: 415.3600 [M-H]$^-$ (Calcd for $C_{33}H_{55}O_3$: 415.3212).

Icosy *trans*-p-coumarate (2): ^{1}H-NMR (pyridine-d_5, 400 MHz) δ_H : 0.89 (3H, t, J=6.9 Hz, Me-20′), 1.27— 1.41 (34H, m, H_2-3′— H_2-19′), 1.73 (2H, m, H_2-2′), 4.31 (2H, t, J=6.9 Hz, H_2-1′), 6.60 (1H, d, J=15.9 Hz, H-8), 7.14 (2H, d, J=8.4 Hz, H-3,5), 7.59 (2H, d, J= 8.4 Hz, H-2,6), 7.95 (1H, d, J=15.9 Hz, H-7). ^{13}C-NMR (pyridine-d_5, 100 MHz) δ_C : 14.1 (C-20′), 22.7 (C-19′), 26.1 (C-18′), 29.0, 29.4 — 29.8 (C-3′— C-17′), 31.9 (C-2′), 64.3 (C-1′), 115.0 (C-8), 116.0 (C-3,6), 125.8 (C-1), 130.4 (C-2,6), 144.9 (C-7), 161.3 (C-4), 167.2 (C-9). HR-ESI-MS (negative-ion mode) m/z: 443.3929 [M-H]$^-$ (Calcd for $C_{29}H_{47}O_3$: 444.3525).

Docosyl *trans-p*-coumarate (3): ^{1}H-NMR (pyridine-d_5, 400 MHz) δ_H : 0.89 (3H, t, J=6.9 Hz, Me-22′), 1.27— 1.41 (38H, m, H_2-3′— H_2-21′), 1.73 (2H, m, H_2-2′), 4.31 (2H, t, J=6.9 Hz, H_2-1′), 6.60 (1H, d, J=15.9 Hz, H-8), 7.14 (2H, d, J=8.4 Hz, H-3,5), 7.59 (2H, d, J= 8.4 Hz, H-2,6), 7.95 (1H, d, J=15.9 Hz, H-7). ^{13}C-NMR (pyridine-d_5, 100 MHz) δ_C : 14.1 (C-22′), 22.7 (C-21′), 26.1 (C-20′), 29.0, 29.4 — 29.8 (C-3′— C-19′), 31.9 (C-2′), 64.3 (C-1′), 115.0 (C-8), 116.0 (C-3,6), 125.8 (C-1), 130.4 (C-2,6), 144.9 (C-7), 161.3 (C-4), 167.2 (C-9). HR-ESI-MS (negative-ion mode) m/z: 471.4265 [M-H]$^-$ (Calcd for $C_{31}H_{51}O_3$: 471.3838).

Tetracosyl *trans-p*-coumarate (4): ^{1}H-NMR (pyridine-d_5, 400 MHz) δ_H : 0.87 (3H, t, J=6.9 Hz, Me-24′), 1.27— 1.41 (42H, m, H_2-3′— H_2-19′), 1.73 (2H, m, H_2-2′), 4.30 (2H, t, J=6.9 Hz, H_2-1′), 6.62 (1H, d, J=15.9 Hz, H-8), 7.15 (2H, d, J=8.4 Hz, H-3,5), 7.57 (2H, d, J= 8.4 Hz, H-2,6), 7.96 (1H, d, J=15.9 Hz, H-7). ^{13}C-NMR (pyridine-d_5, 100 MHz) δ_C : 14.1 (C-24′), 22.7 (C-23′), 26.1 (C-22′), 29.0, 29.4 — 29.8 (C-3′— C-21′), 31.9 (C-2′), 64.3 (C-1′), 115.0 (C-8), 116.6 (C-3,6), 125.9 (C-1), 130.5 (C-2,6), 144.9 (C-7), 161.3 (C-4), 167.2 (C-9). HR-ESI-MS (negative-ion mode) m/z: 499.4589 [M-H]$^-$ (Calcd for $C_{33}H_{55}O_3$: 499.4151).

Santonin (5): ^{1}H-NMR ($CDCl_3$, 400 MHz) δ_H: 1.02 (3H, d, J=6.9 Hz, Me-13); 1.13 (3H, s, Me-14); 1.86 (3H, s, Me-15); 1.63 (2H, m, H-9); 1.82 (2H, m, H-8); 2.29 (1H, m, H-11); 4.66 (1H, d, d, J=11.0 Hz, H-6); 5.98 (1H, d, J=9.8 Hz, H-2); 6.55 (1H, d, J=9.8 Hz, H-1); ^{13}C-NMR ($CDCl_3$, 100 MHz) δ_C: 10.5 (C-15), 12.1 (C-13), 22.4 (C-8), 24.6 (C-14), 37.4 (C-9), 40.3 (C-11), 41.1 (C-10), 53.1 (C-7), 80.9 (C-6), 125.1 (C-2), 127.6 (C-4), 151.3 (C-5), 155.1 (C-1), 177.4 (C-12), 185.8 (C-3); HR-ESI-MS (positive-ion mode) m/z: 247.1351 [M+H]$^+$ (calcd for $C_{15}H_{19}O_3$: 246.1256), 269.1175 [M+Na]$^+$ (calcd for $C1_5H_{18}O_3Na$: 269.1154), 515.2451 [2M+Na]$^+$ (calcd for $C_{30}H_{36}O_6Na$: 515.2410).

Axillarin (6): ^{1}H-NMR (pyridine-d_5, 400 MHz) δ_H: 3.92 (3H, s, OMe-3), 3.99 (3H, s, OMe-6), 6.79 (IH, s, H-8), 7.35 (1H, d, J=8.4 Hz, H-5′), 7.81(1H, dd, J=8.4, 2.2 Hz, H-6′), 8.18 (1H, d, J=2.2 Hz, H-2′); ^{13}C-NMR (pyridine-d_5, 100 MHz) δ_C: 59.7 (C-6), 60.2 (C-3), 94.6 (C-8), 105.6 (C-10), 116.4 (C-2′), 116.5 (C-6′), 121.4 (C-5′), 122.0 (C-1′), 132.0 (C-6), 138.2 (C-3), 147.0 (C-3′), 150.5 (C-4′), 152.6 (C-9), 153.5 (C-5), 156.4 (C-2), 158.6 (C-7), 179.0 (C-4). HR-ESI-MS (negative-ion mode) m/z: 345.0918 [M-H]$^-$ (Calcd for $C_{17}H_{13}O_8$: 345.0610).

Quercetin 3-*O*-methyl ether (7): ^{1}H-NMR (pyridine-d_5, 400 MHz) δ_H: 3.91 (3H, s, OMe-3), 6.72 (IH, d, J= 1.9 Hz, H-8), 6.74 (IH, d, J= 1.9 Hz, H-6), 7.35 (1H, d, J=8.4 Hz, H-5′), 7.81(1H, dd, J=8.4, 2.2 Hz, H-6′), 8.18 (1H, d, J=2.2 Hz, H-2′); ^{13}C-NMR (pyridine-d_5, 100 MHz) δ_C: 59.7 (C-3), 94.3 (C-8), 99.4 (C-6), 105.2 (C-10), 116.4 (C-2′), 116.5 (C-6′), 121.4

(C-5′), 121.9 (C-1′), 138.5 (C-3), 146.9 (C-3′), 150.5 (C-4′), 156.4 (C-2), 157.3 (C-9), 162.6 (C-5), 165.6 (C-7), 178.8 (C-4). HR-ESI-MS (negative-ion mode) m/z: 315.0593 $[M-H]^-$ (Calcd for $C_{16}H_{11}O_7$: 315.0505).

Luteolin (**8**): ^{1}H-NMR (CD_3OD, 400 MHz) δ_H: 6.18 (IH, d, J= 1.8 Hz, H-8), 6.37 (IH, d, J= 1.8 Hz, H-6), 6.44 (1H, s, H-3), 6.89 (1H, d, J=8.2 Hz, H-5′), 7.50 (1H, dd, J=8.2, 2.2 Hz, H-6′), 7.82 (1H, d, J=2.2 Hz, H-2′); ^{13}C-NMR (CD_3OD, 100 MHz) δ_C: 94.7 (C-8), 99.8 (C-6), 103.4 (C-3), 105.8 (C-10), 114.1 (C-2′), 116.4 (C-6′), 120.8 (C-5′), 122.9 (C-1′), 146.4 (C-3′), 149.9 (C-4′), 157.9 (C-9), 163.0 (C-7), 165.9 (C-2), 165.9 (C-5), 179.9 (C-4). HR-ESI-MS (negative-ion mode) m/z: 285.0487 $[M-H]^-$ (Calcd for $C_{15}H_9O_6$: 285.0399).

Quercetin (**9**): ^{1}H-NMR (pyridine-d_5, 400 MHz) δ_H: 6.74 (IH, d, J= 2.1 Hz, H-8), 6.78 (IH, d, J= 2.1 Hz, H-6), 7.40 (1H, d, J=8.5 Hz, H-5′), 8.10 (1H, dd, J=8.5, 2.2 Hz, H-6′), 8.81 (1H, d, J=2.2 Hz, H-2′); ^{13}C-NMR (pyridine-d_5, 100 MHz) δ_C: 94.1 (C-8), 99.0 (C-6), 104.2 (C-10), 116.3 (C-2′), 116.4 (C-6′), 120.8 (C-5′), 125.2 (C-1′), 137.7 (C-3), 146.9 (C-3′), 147.5 (C-4′), 157.1 (C-2), 157.2 (C-9), 162.2 (C-5), 165.3 (C-7), 177.0 (C-4). HR-ESI-MS (negative-ion mode) m/z: 301.0712 $[M-H]^-$ (Calcd for $C_{15}H_9O_7$: 301.0348).

***p*-Coumaric acid** (**10**): ^{1}H-NMR (CD_3OD, 400 MHz) δ_H: 6.33 (1H, d, J=15.8 Hz, H-8), 6.78 (2H, d, J=8.6 Hz, H-3,5), 7.37 (2H, d, J= 8.6 Hz, H-2,6), 7.85 (1H, d, J=15.8 Hz, H-7); ^{13}C-NMR (CD_3OD, 100 MHz) δ_C: 115.3 (C-8), 116.6 (C-3,6), 125.8 (C-1), 130.2 (C-2,6), 141.7 (C-7), 161.3 (C-4), 167.2 (C-9). HR-ESI-MS (negative-ion mode) m/z: 163.0601 $[M-H]^-$ (Calcd for $C_9H_7O_3$: 163.0395).

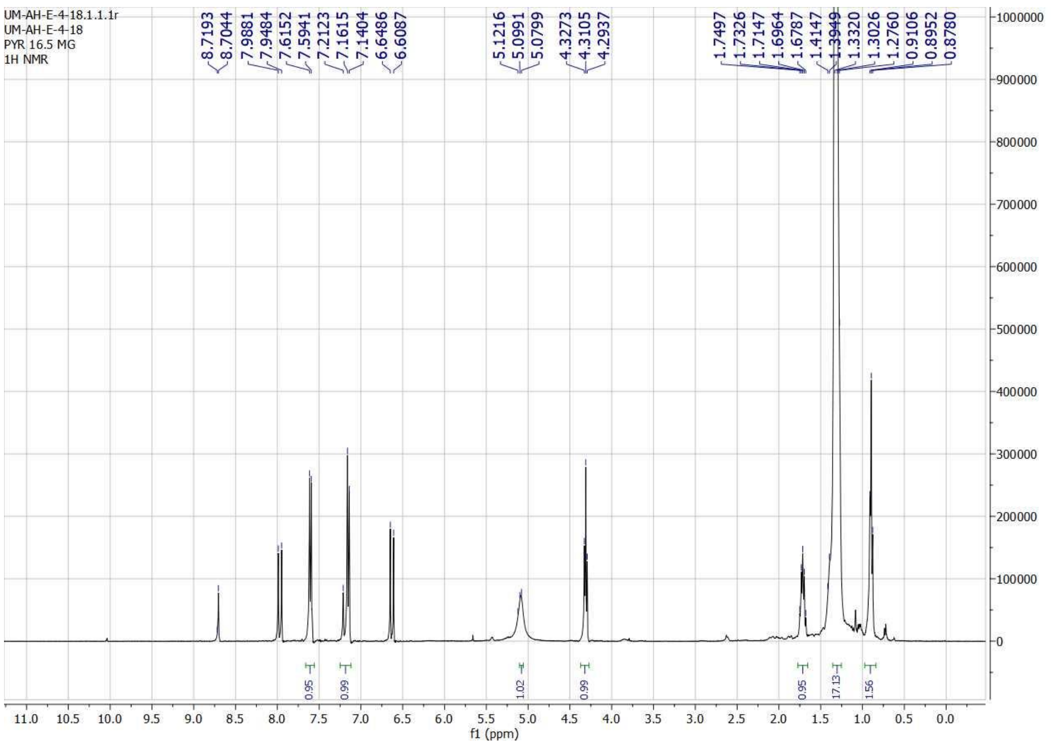

Figure S1. ^{1}H NMR spectrum of compound **2-4** (C_5D_5N, 400 MHz).

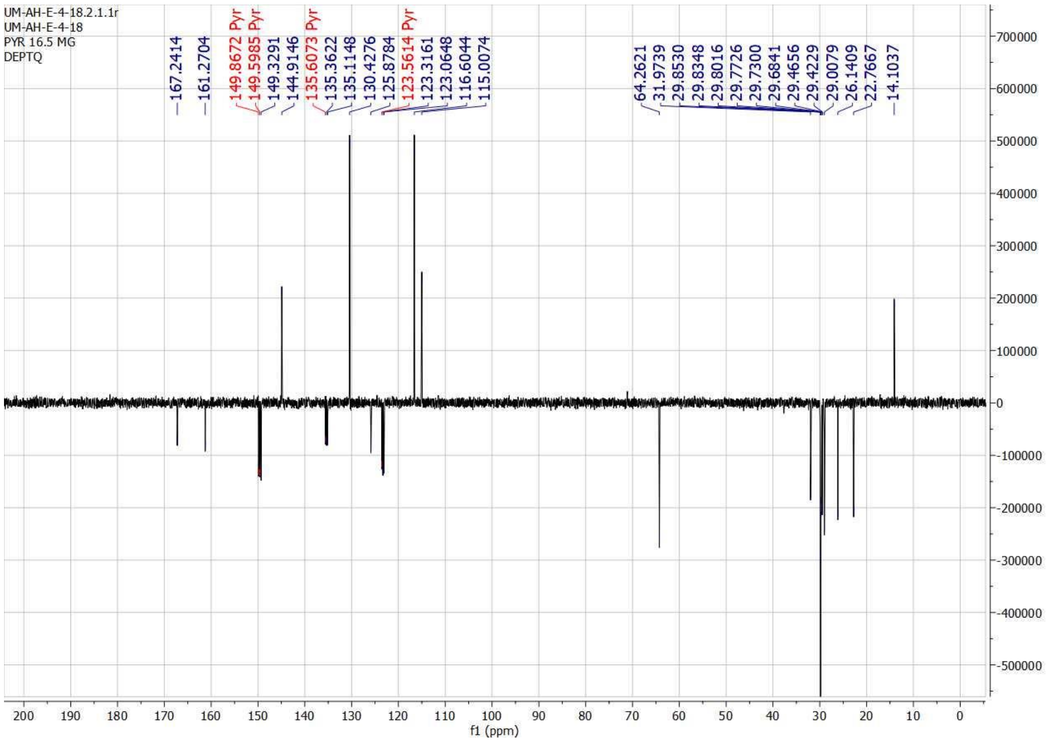

Figure S2. DEPTQ 135 NMR spectrum of compound **2-4** (C_5D_5N, 100 MHz).

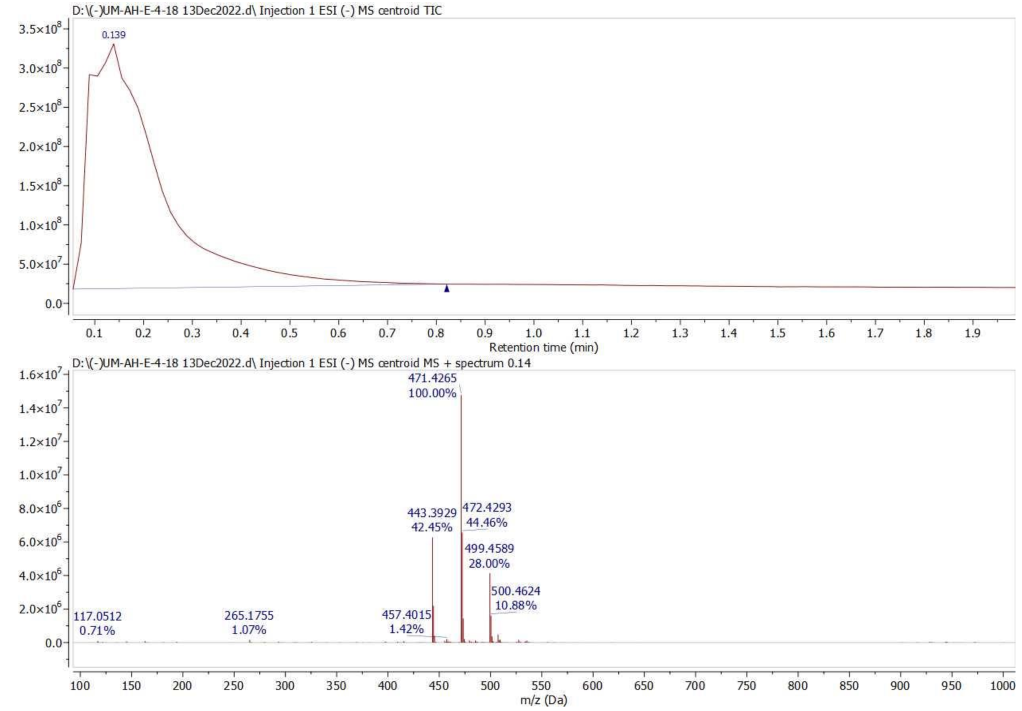

Figure S3. HR-ESI-MS spectrum of compounds **2-4.**

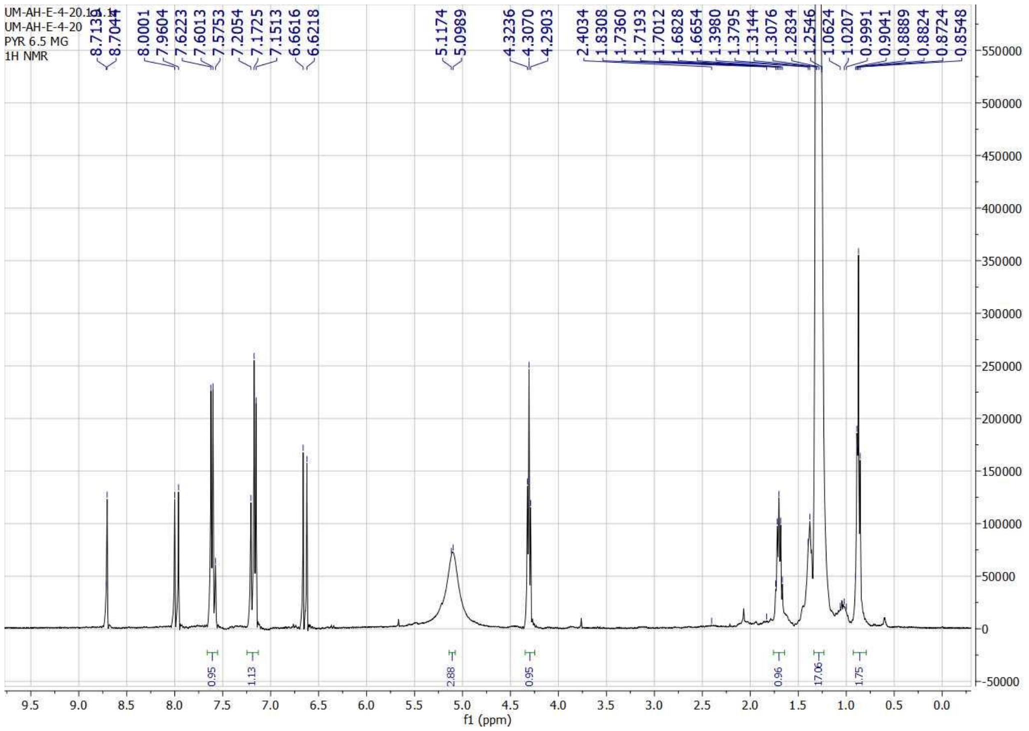

Figure S4. ^{1}H NMR spectrum of compounds **1 - 4** (C_5D_5N, 400 MHz).

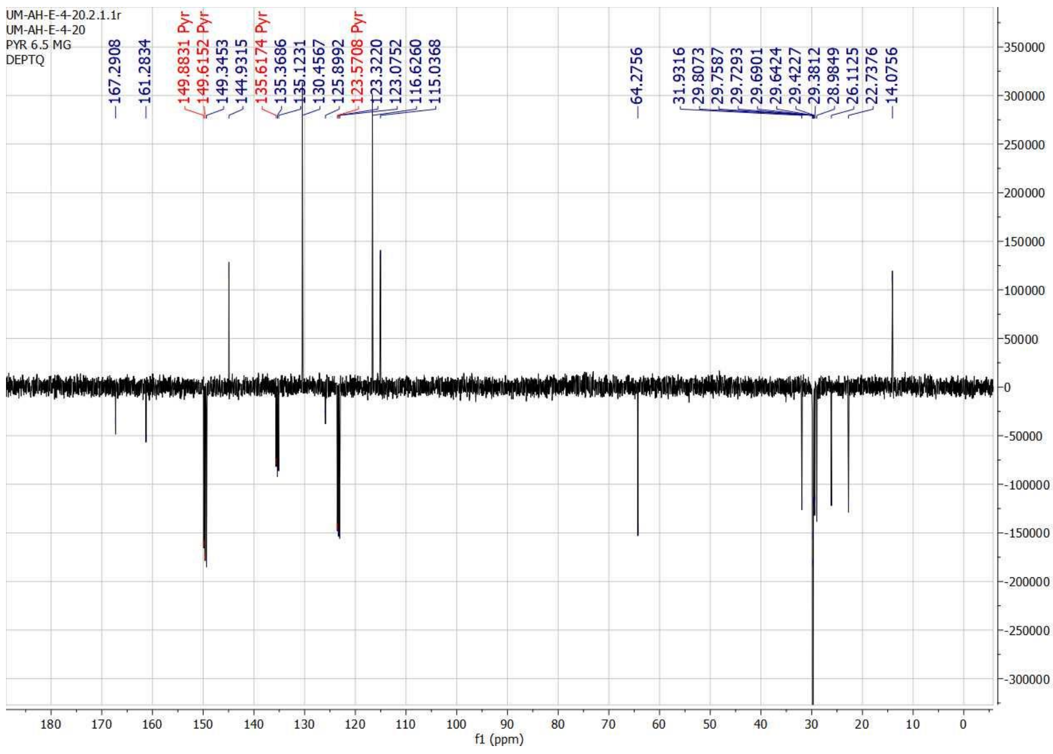

Figure S5. DEPTQ 135 NMR spectrum of compounds **1 - 4** (C_5D_5N, 100 MHz).

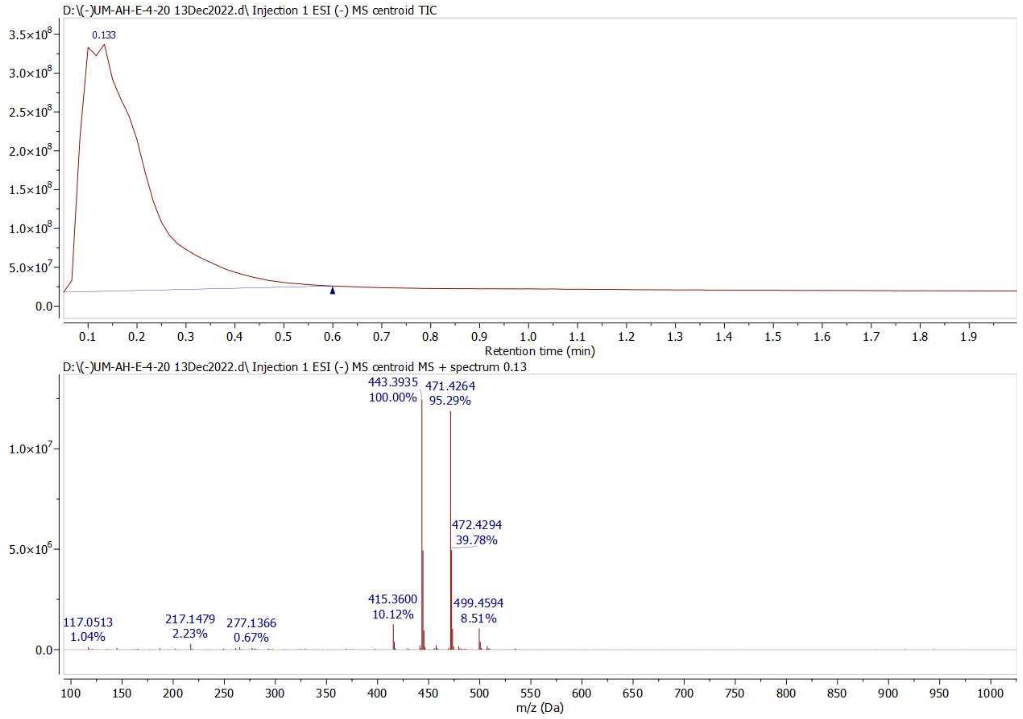

Figure S6. HR-ESI-MS spectrum of compounds **1 - 4.**

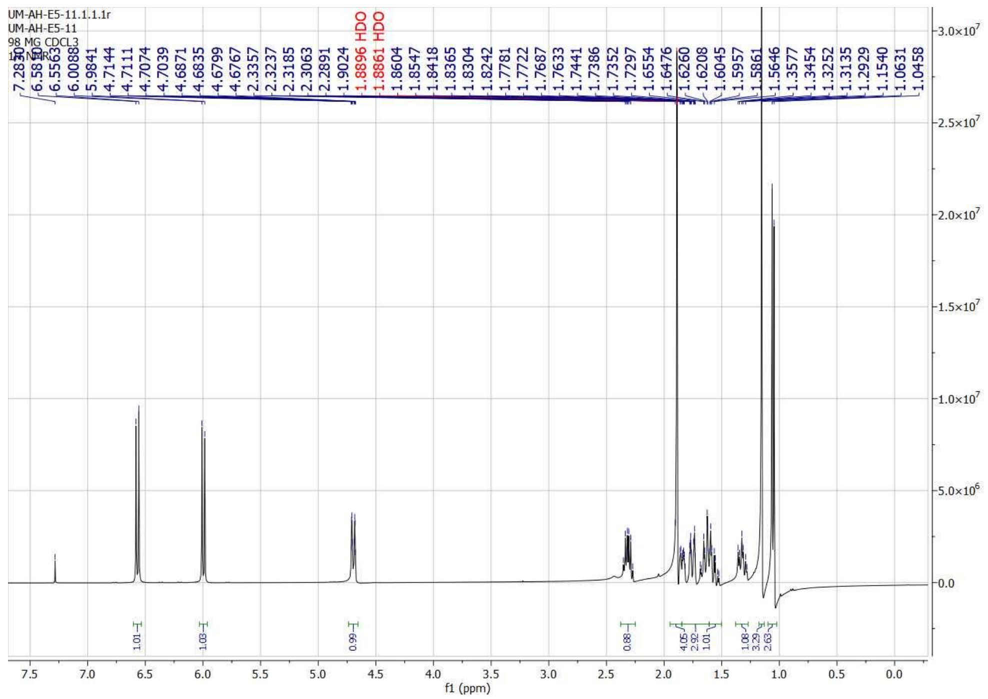

Figure S7. ^{1}H NMR spectrum of compound **5** ($CDCl_3$, 400 MHz).

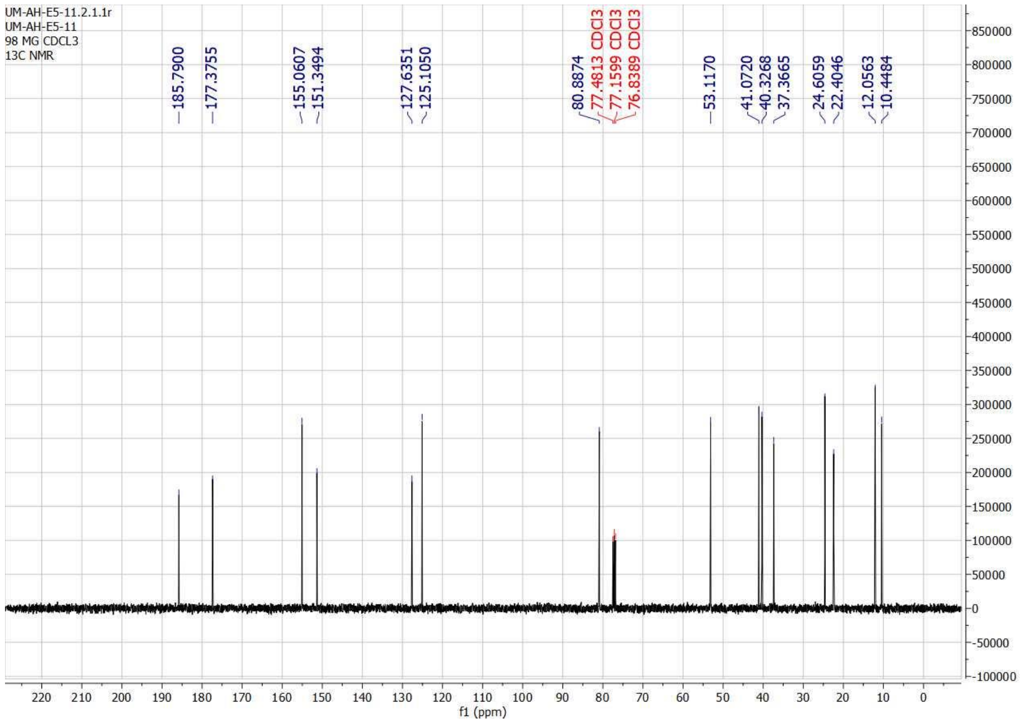

Figure S8. ^{13}C NMR spectrum of compound **5** ($CDCl_3$, 100 MHz).

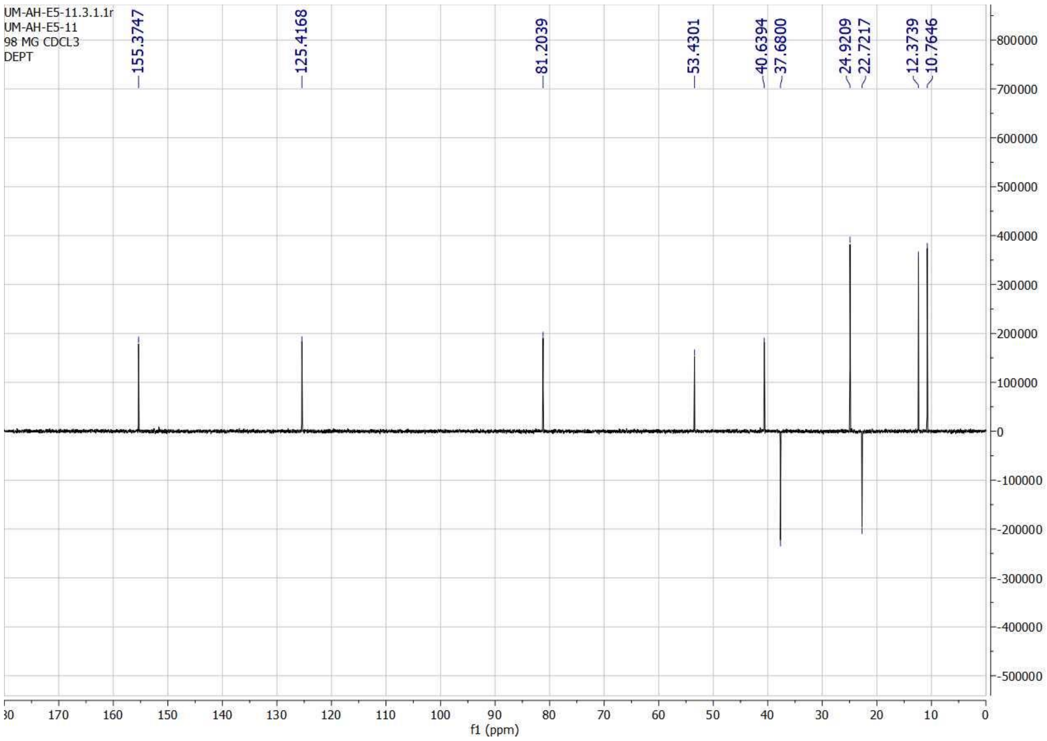

Figure S9. DEPT 135 NMR spectrum of compound **5** ($CDCl_3$, 100 MHz).

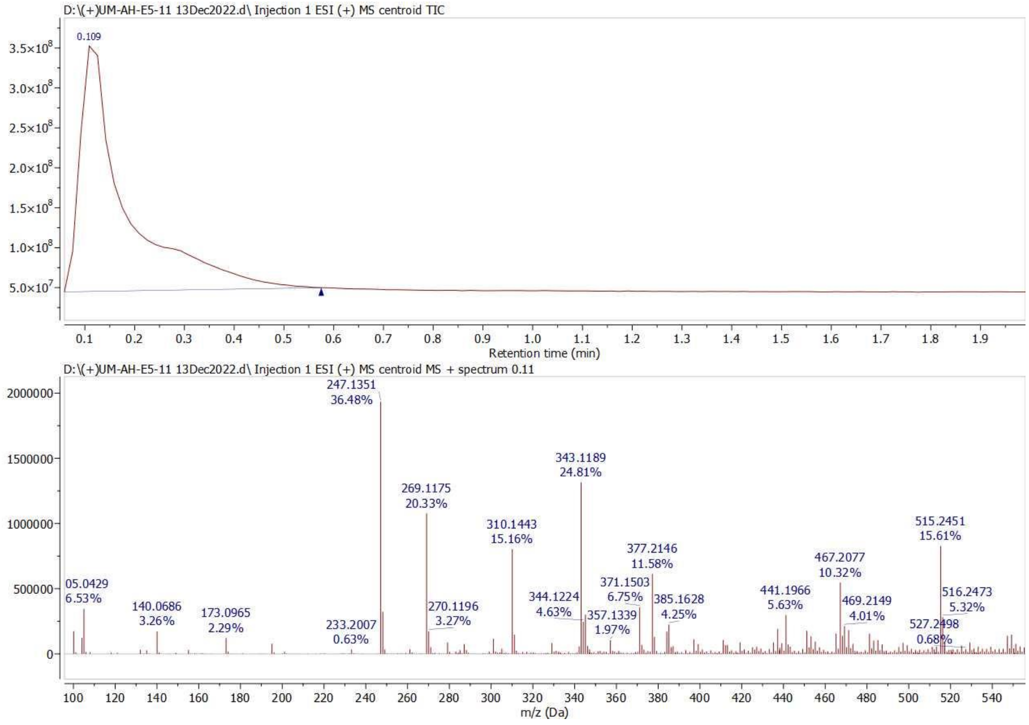

Figure S10. HR-ESI-MS spectrum of compound **5.**

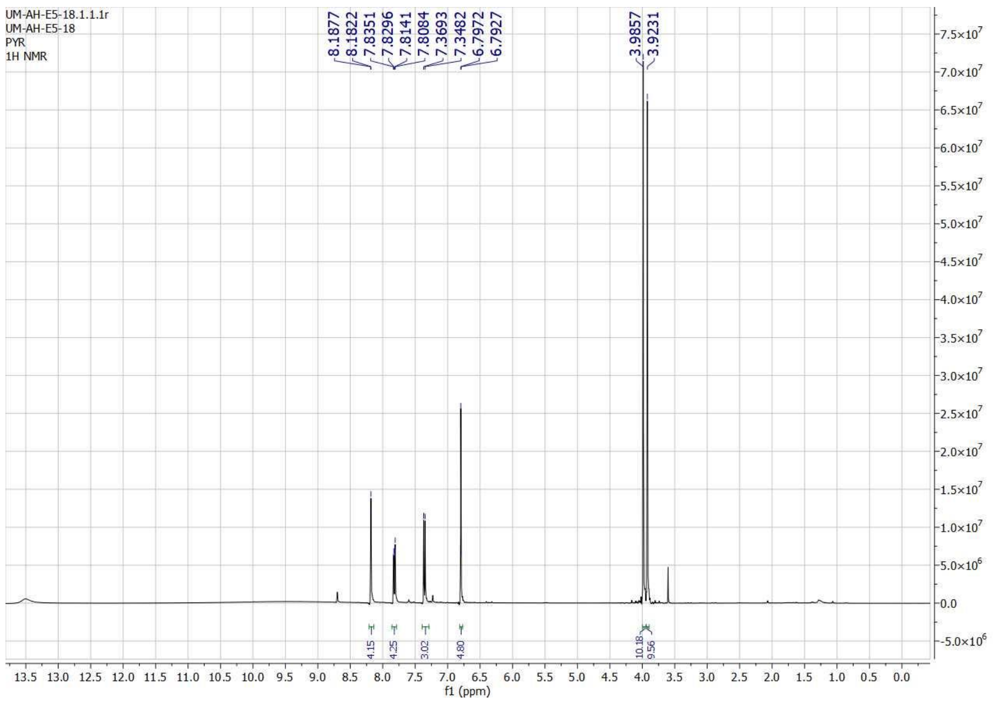

Figure S11. ^{1}H NMR spectrum of compound **6** (C_5D_5N, 400 MHz).

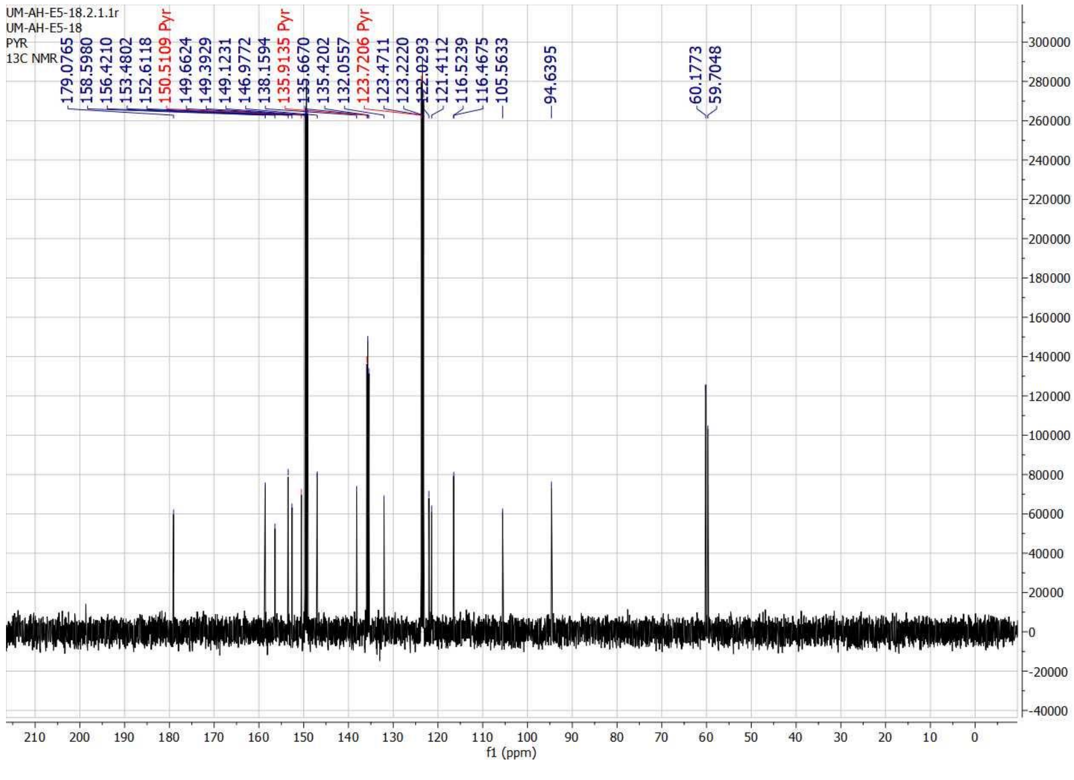

Figure S12. ^{13}C NMR spectrum of compound **6** (C_5D_5N, 100 MHz).

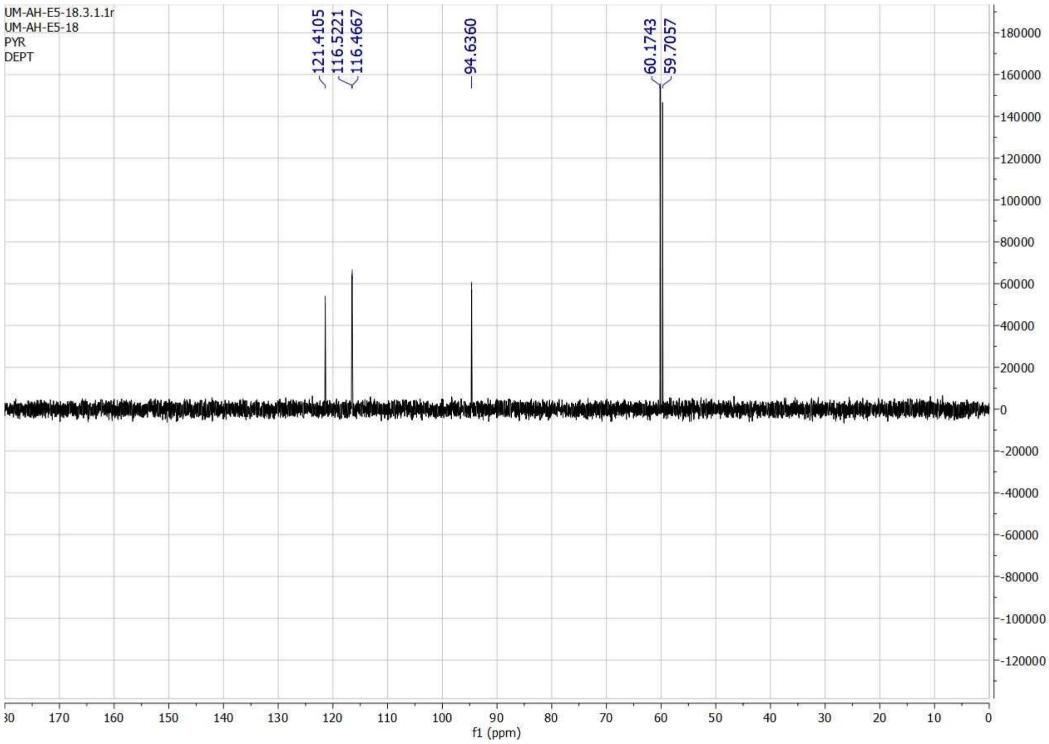

Figure S13. DEPT 135 NMR spectrum of compound **6** (C_5D_5N,100 MHz).

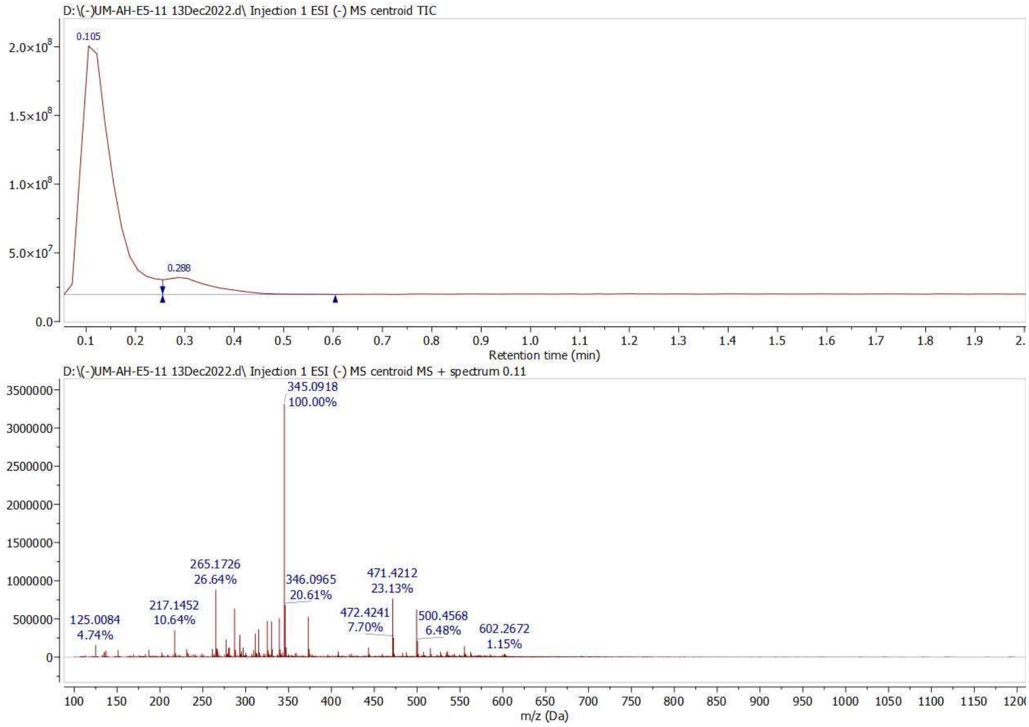

Figure S14. HR-ESI-MS spectrum of compound **6.**

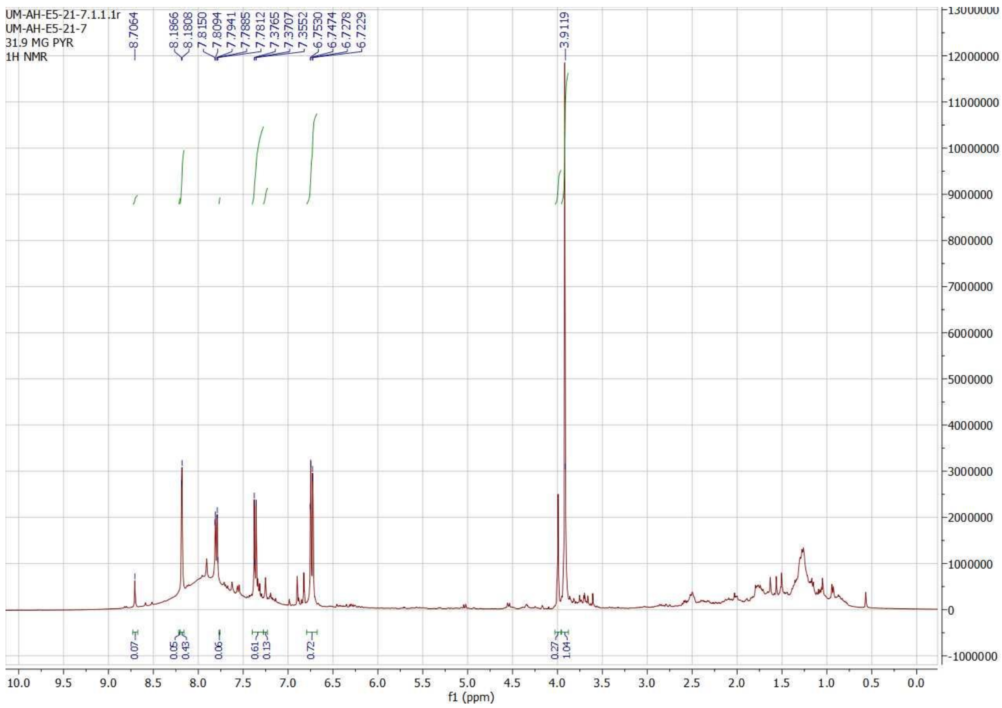

Figure S15. ^{1}H NMR spectrum of compound **7** (C_5D_5N, 400 MHz).

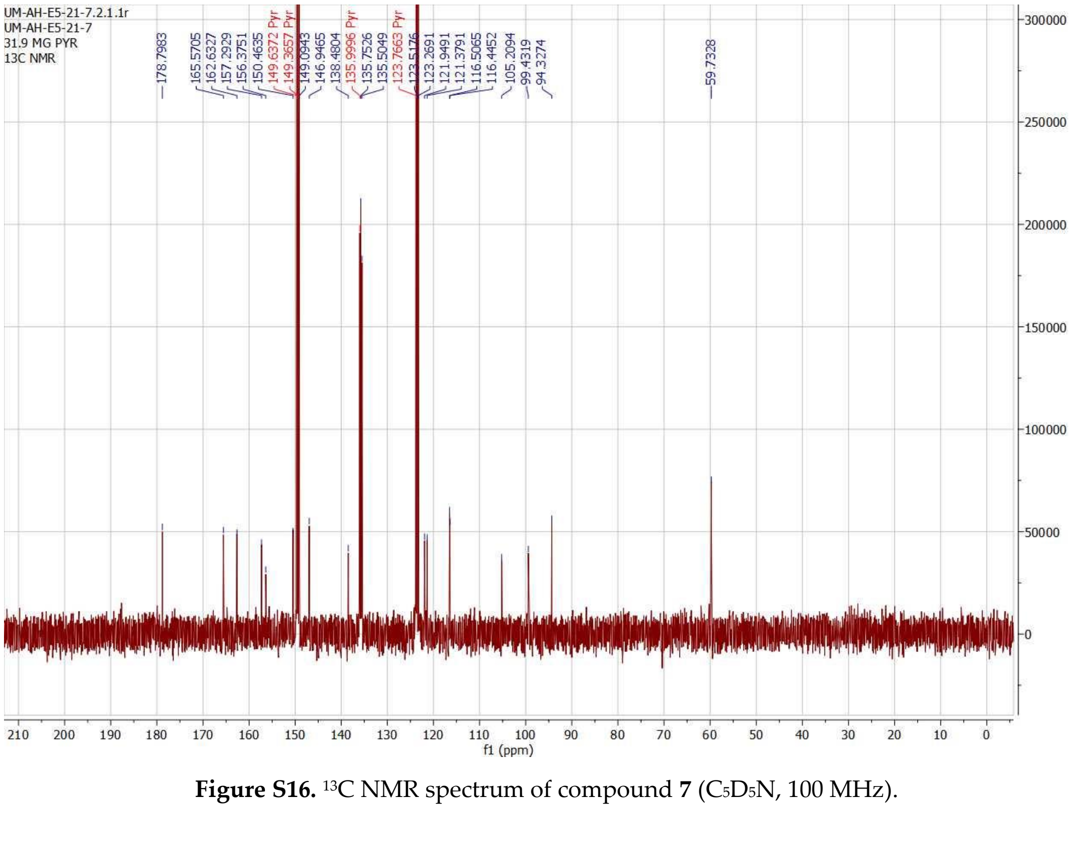

Figure S16. ^{13}C NMR spectrum of compound **7** (C_5D_5N, 100 MHz).

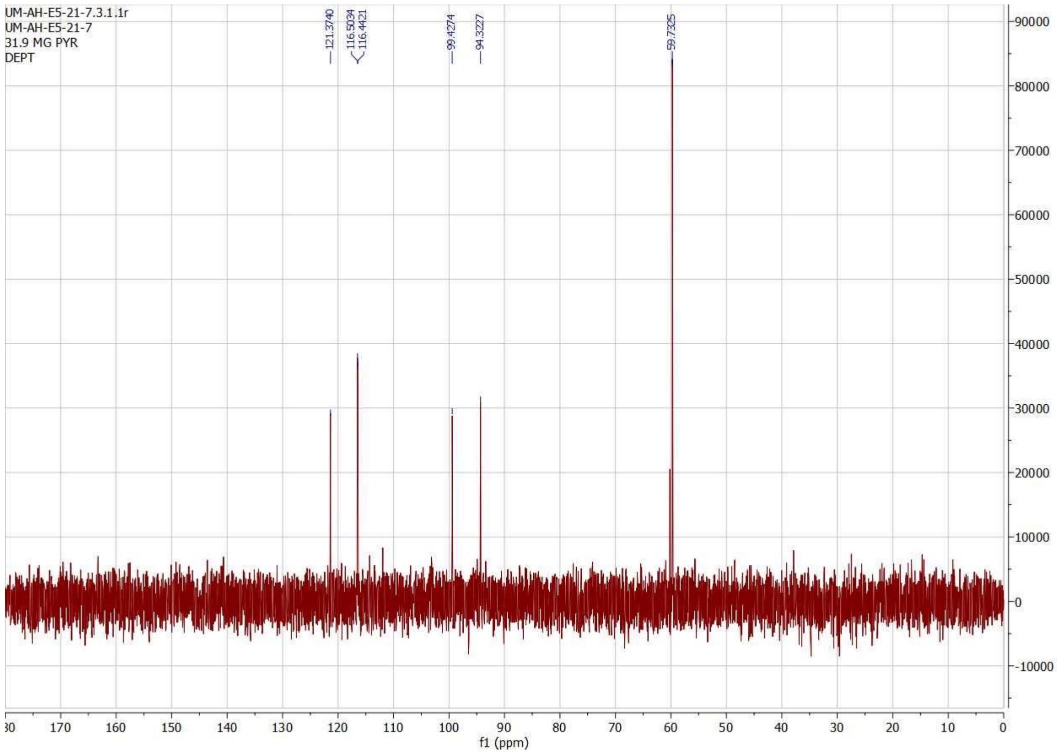

Figure S17. DEPT 135 NMR spectrum of compound **7** (C_5D_5N, 100 MHz).

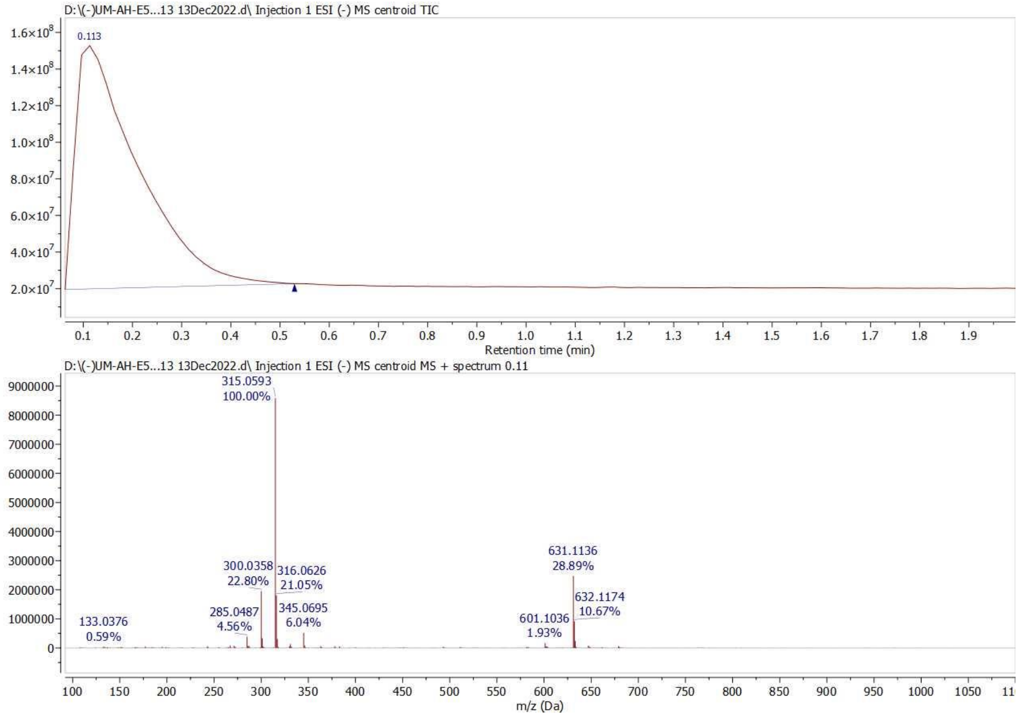

Figure S18. HR-ESI-MS spectrum of compound **7.**

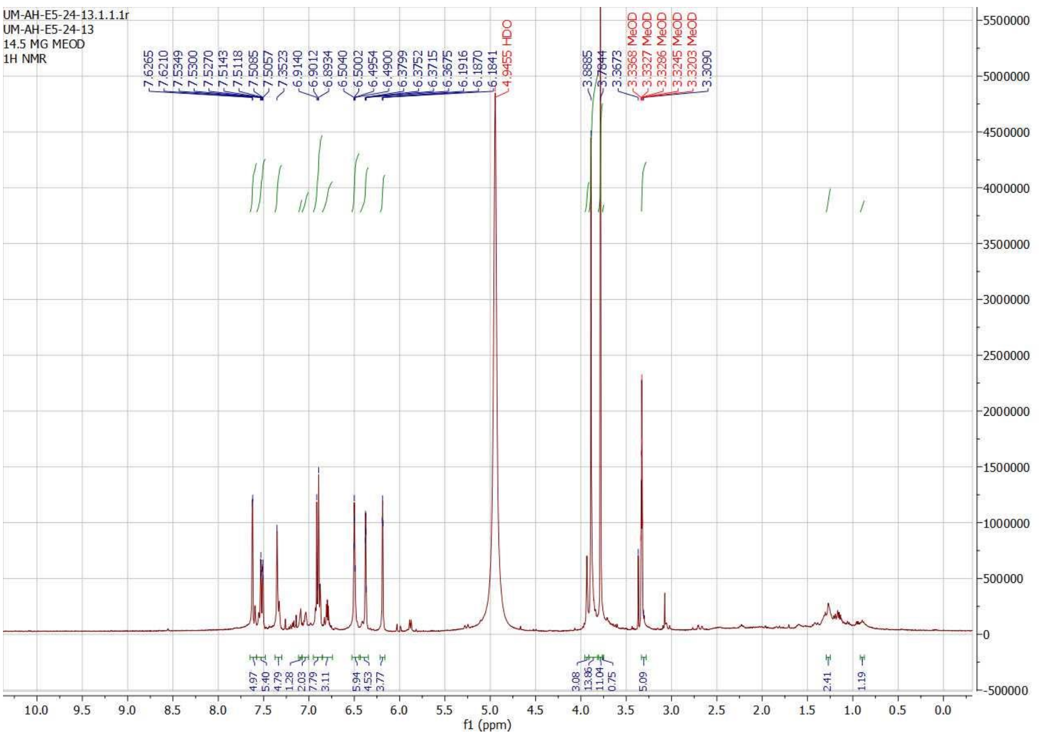

Figure S19. ^{1}H NMR spectrum of compound **8** (CD_3OD, 400 MHz).

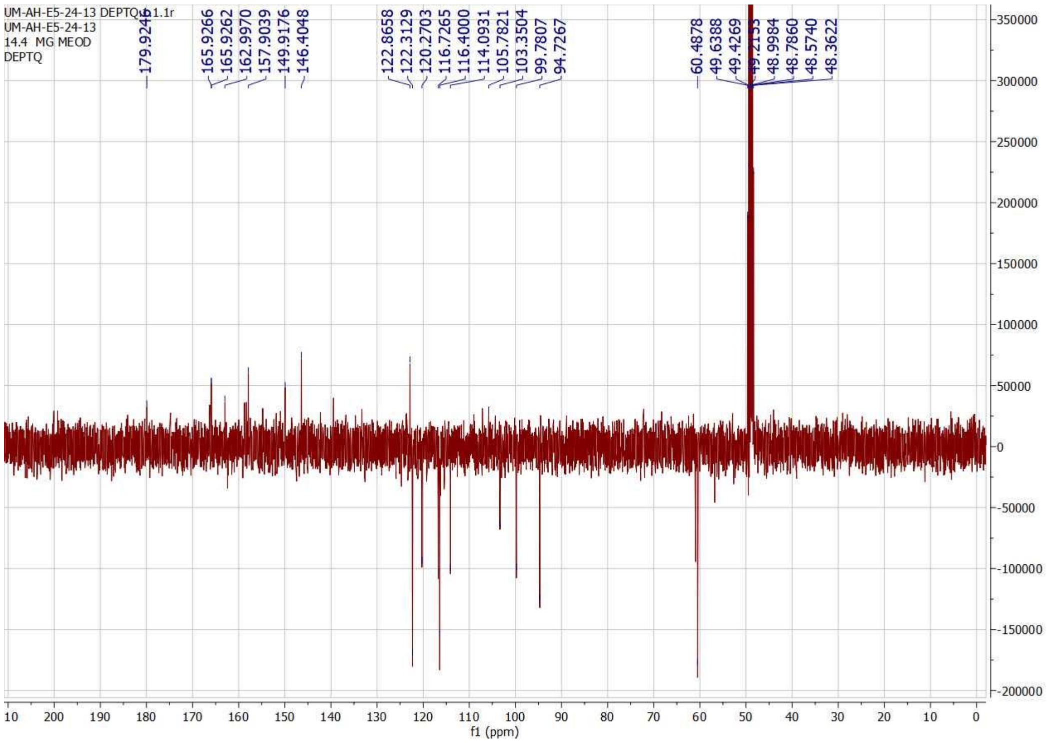

Figure S20. DEPTQ 135 NMR spectrum of compound **8** (CD_3OD, 100 MHz).

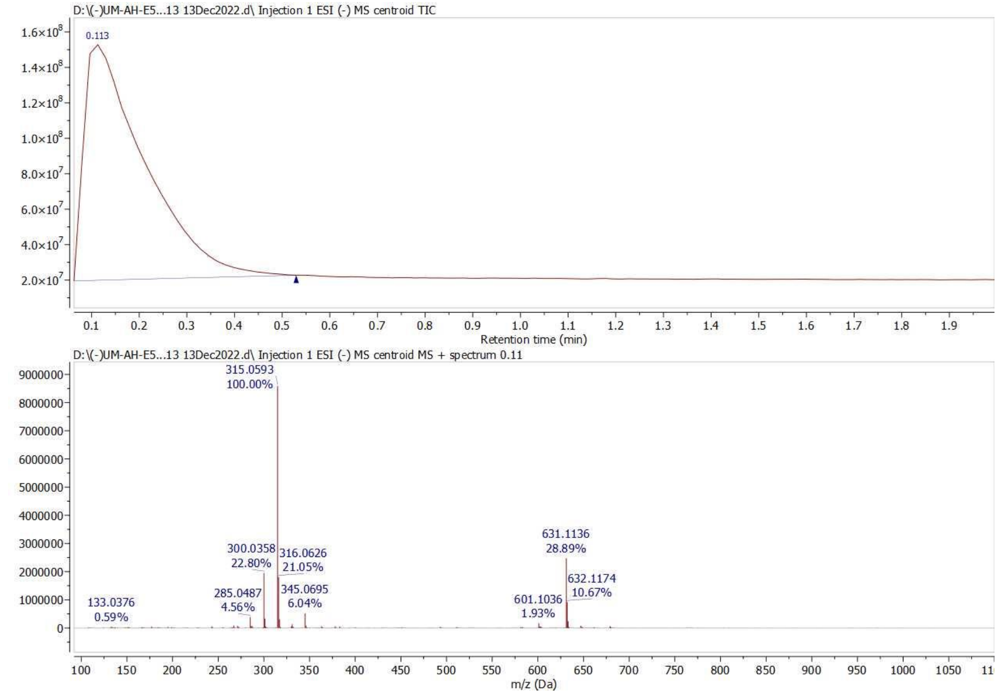

Figure S21. HR-ESI-MS spectrum of compound **8.**

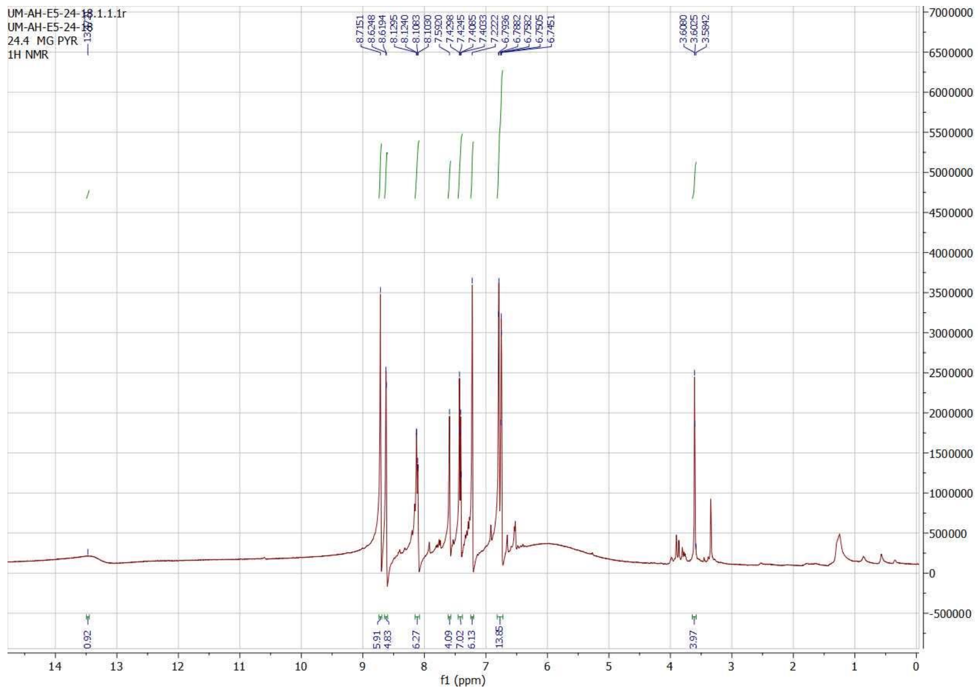

Figure S22. ^{1}H NMR spectrum of compound **9** (C_5D_5N, 400 MHz).

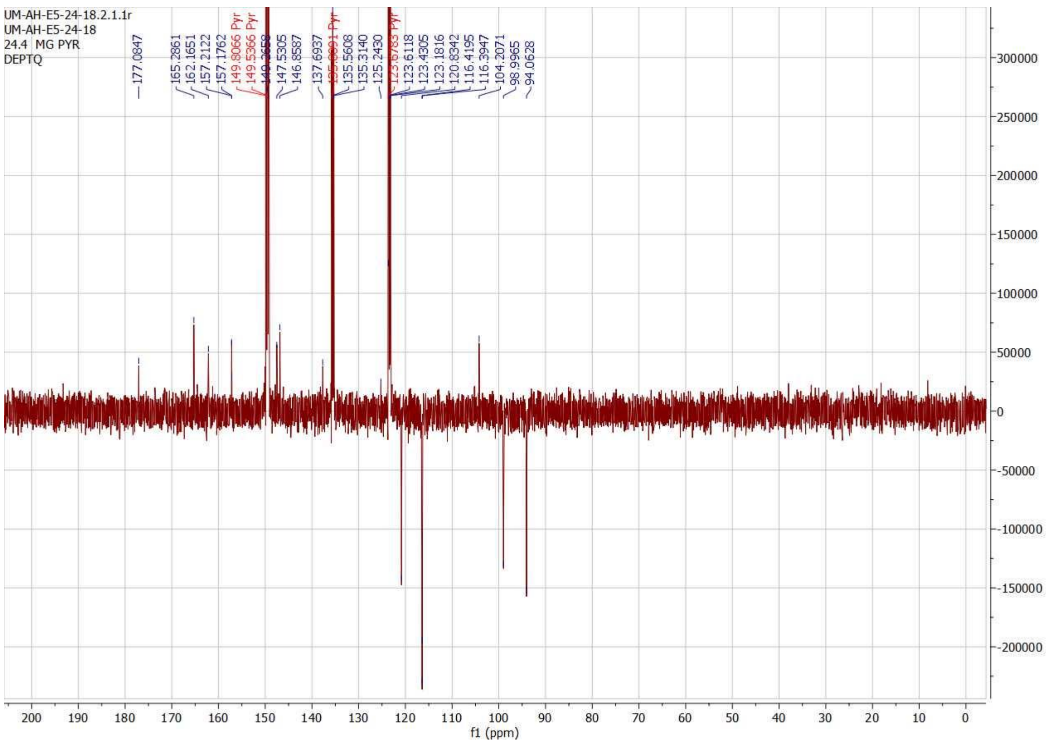

Figure S23. DEPTQ 135 NMR spectrum of compound **9** (C_5D_5N, 100 MHz).

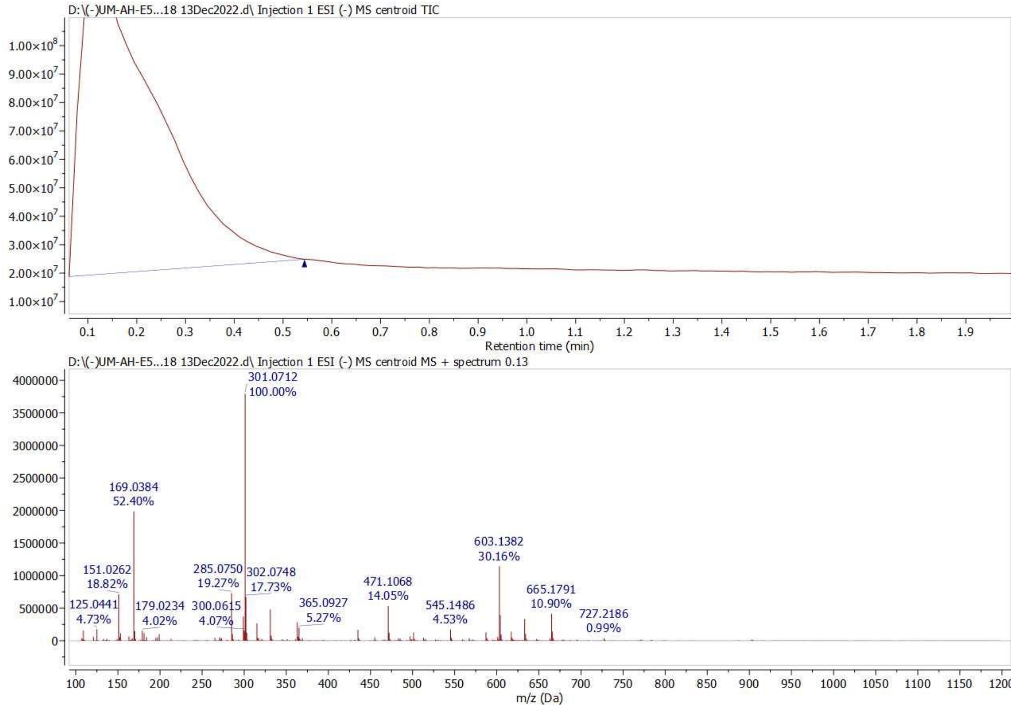

Figure S24. HR-ESI-MS spectrum of compound **9.**

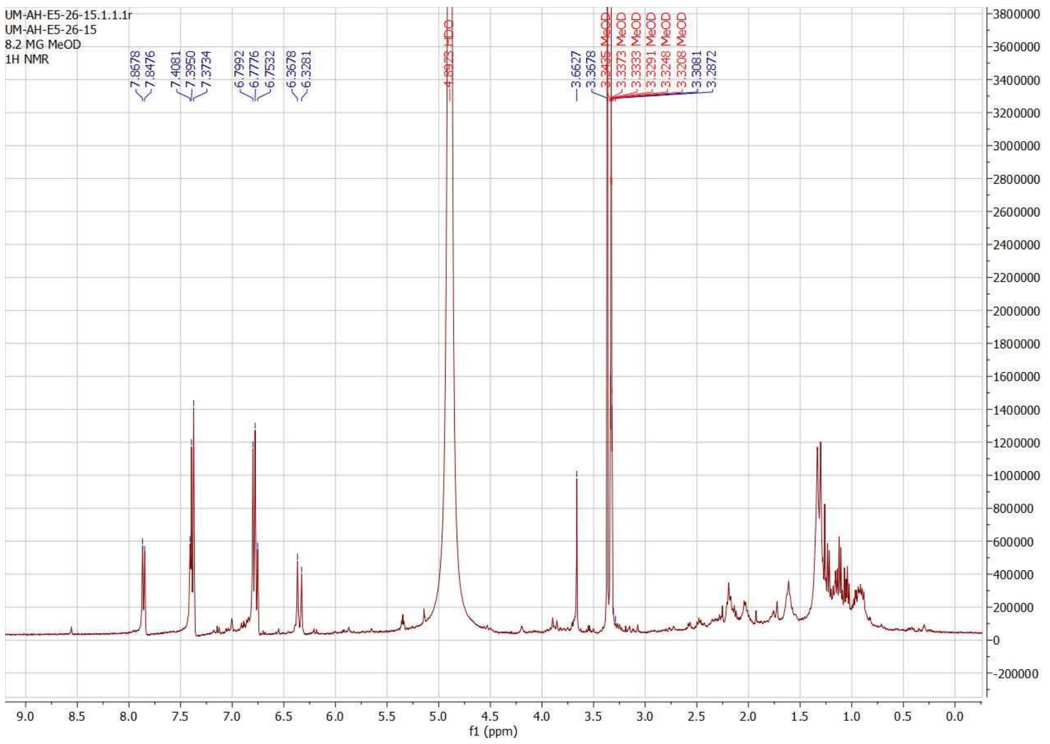

Figure S25. ^{1}H NMR spectrum of compound **10** (CD_3OD, 400 MHz).

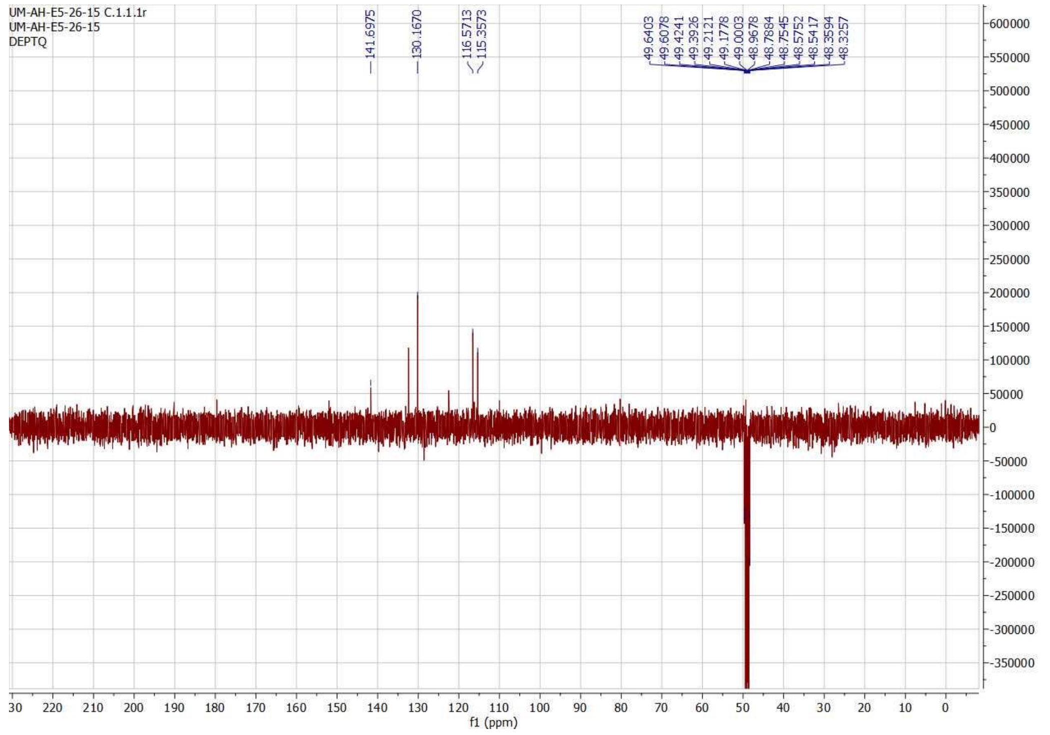

Figure S26. DEPTQ 135 NMR spectrum of compound **10** (CD_3OD, 100 MHz).

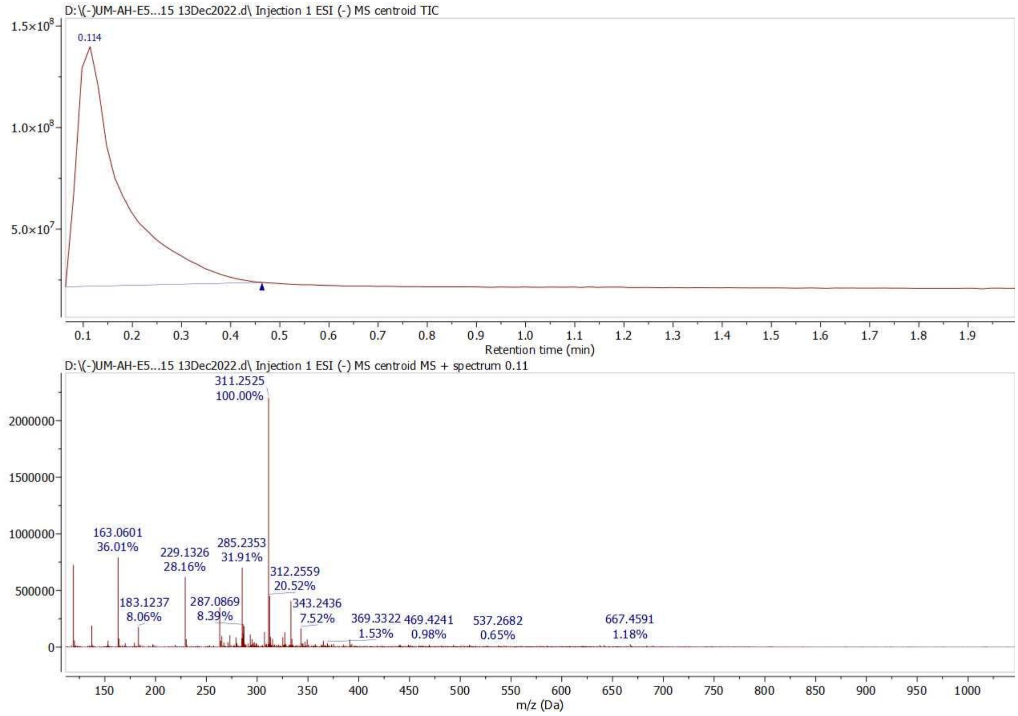

Figure S27. HR-ESI-MS spectrum of compound **10.**
